# Revisiting Infanticide in Non‐Human Primates Reveals a Similar Likelihood of Male and Female Perpetrators

**DOI:** 10.1002/ajp.70132

**Published:** 2026-03-23

**Authors:** Tatiani G. Albert, Nicola Schiel, Marcelo A. Ramos, Antonio Souto

**Affiliations:** ^1^ Departamento de Biologia Universidade Federal Rural de Pernambuco Recife Brasil; ^2^ Departamento de Biologia Universidade de Pernambuco ‐ Campus Mata Norte Nazaré da Mata Brasil; ^3^ Departamento de Zoologia Universidade Federal de Pernambuco Recife Brasil

**Keywords:** direct infanticide, exploitation, indirect infanticide, parental manipulation, resource competition, sex bias

## Abstract

Infanticide is understood as any direct or indirect behavior that fatally harms an infant, regardless of whether the perpetrator gains benefits. In non‐human primates, males are frequently identified as the perpetrators. Classical studies categorized behaviors like “abuse”, “fatal neglect”, “kidnapping”, and “aunting to death” as forms of infanticide when they resulted in infant death. However, in more recent literature, some of these behaviors are excluded from classifications of infanticide without clear justification, particularly those involving female‐related lethal actions. Therefore, we conducted a systematic review spanning 54 years to investigate potential sex biases in primate infanticide literature. Our findings show that female actions leading to infant death are often labeled with terms like “abuse” and “fatal neglect”. In contrast, similar male behaviors are consistently classified as infanticide. As a result, infanticide by non‐human primate females has been systematically underreported. Classifying all lethal behaviors by females toward infants as infanticide eliminates the sex difference in the frequency of such acts. Our study shows that being consistent with the original definition of infanticide for non‐human primates provides a more accurate understanding of infanticide in these animals. Thus, we strongly recommend adhering to the infanticide definition, which integrates the recorded behaviors into established theoretical frameworks, enabling more comprehensive discussions of primate infanticidal behavior.

## Summary

- Infanticide by non‐human females has been underreported as lethal behaviors are often not classified as infanticide. In contrast, this does not occur when males are the perpetrators, revealing a bias in how these behaviors are categorized.
- Our analyses, drawing on the classical understanding of infanticide, which incorporates abuse, fatal neglect, kidnapping, and aunting to death, reveal no statistically significant differences in infanticide levels committed by males and females.
- By using infant death as the definitive outcome measure, following classical studies on the subject, we provide a more comprehensive basis for evaluating infanticide across primate species.

## Graphical abstract

This review synthesizes 54 years of data on infanticide in non‐human primates, revealing a sex bias in terminology rather than in behavior. Applying Hrdy's (1979) and Hausfater & Hrdy's (1984) definitions reduces potential sex‐based bias and clarifies that both males and females engage comparably in lethal infant‐directed acts.

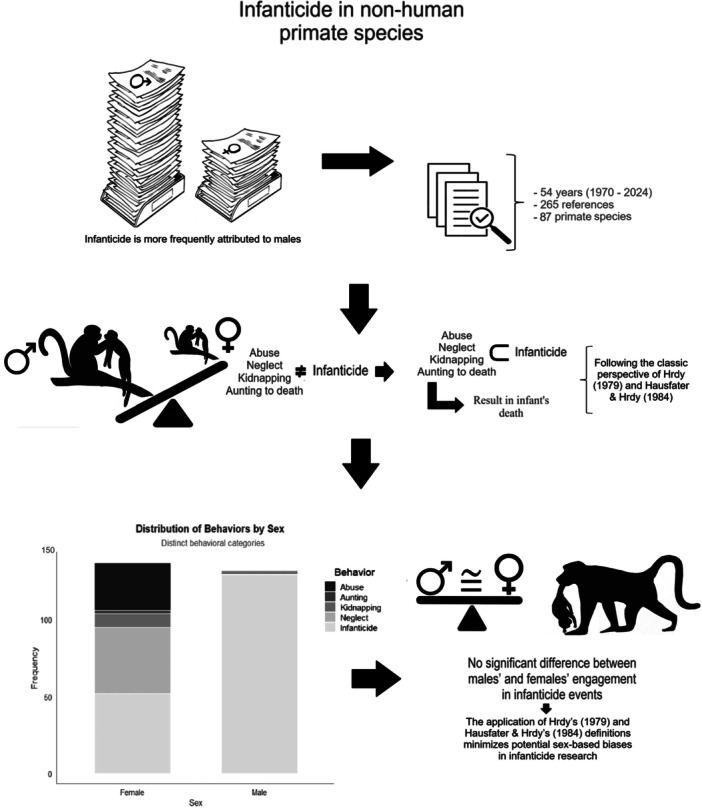

## 1 Introduction

Infanticide, the killing of young offspring by a conspecific, is a pervasive and multifaceted phenomenon documented across numerous non‐human primate species (e.g., Digby 2009; Lukas and Huchard 2014; 2019; Mitani et al. 2012). Numerous studies have carefully documented infanticidal incidents in mammals, and researchers have suggested that males perpetrate most of them (Ebensperger 1998; Lukas and Huchard 2014; 2019; Palombit 2015).

Hrdy (1979) provided foundational work for understanding the many contexts of infanticide, proposing five functional categories: exploitation, resource competition, parental manipulation, sexual selection, and social pathology (e.g., Bezerra et al. 2007; Culot et al. 2011; Nishie and Nakamura 2018). In addition, her comprehensive definition conceptualizes infanticide as any harmful act, including fatal neglect, that causes the death of an infant because of a conspecific's actions, potentially yielding direct or indirect benefits to the perpetrator (Hrdy 1979; Hausfater and Hrdy 1984). Nevertheless, it is also recognized that such behaviors may arise under atypical circumstances, such as social dysfunctions, where no discernible evolutionary advantage is conferred upon the perpetrator (Hausfater and Hrdy 1984; Hrdy 1979). These intricate actions, regardless of intent, highlight the complexity of underlying motivations and behavioral strategies (Hausfater and Hrdy 1984; Hrdy 1979). For further explanations on the causes and functions of infanticide in mammals, see Lukas and Huchard (2014; 2019), Palombit (2015).

Our initial goal was to conduct a general and updated review of infanticide events in non‐human primates. However, during this process, we identified pronounced methodological inconsistencies: certain behaviors observed in females, and directly linked to infant mortality, have often been excluded from infanticide classifications, even though Hrdy's seminal framework (1979) explicitly included them. These behaviors comprise “abuse”, “fatal neglect”, “kidnapping” and “aunting to death”, all of which are relatively common in non‐human primates and widely discussed in the literature (e.g., “abuse”: Brent et al. 2002b; Maestripieri and Carroll 2000; “fatal neglect”: Maestripieri et al. 1997; Maestripieri and Carroll 2000; “kidnapping”: Hrdy 1979; Hsu et al. 2000; “aunting to death”: Hsu et al. 2000; Redman and Schneider 1979). We hypothesize that such exclusions contributed to existing findings suggesting that female non‐human primates engage in fewer acts of infanticide than males (e.g., Lukas and Huchard 2014, 2019; Mitani et al. 2012).

To address our hypothesis that behavioral misclassification may have biased previous interpretations and to provide a more nuanced assessment of sex differences in lethal infant‐directed behaviors, we conducted a comprehensive systematic review of the non‐human primate literature. Beyond compiling records, we examined the possible causes for the terminological discrepancies across studies. By critically examining terminological variations and discussing their possible social underpinnings, our approach refines current understanding of infanticide in both sexes of non‐human primates.

## 2 Methods

We considered infanticide as any harmful behavior that leads to the death of an infant, carried out by conspecific individuals, including the infant's parents (Hausfater and Hrdy 1984; Hrdy 1979). An infant is understood as a young primate dependent on adults for nourishment, protection, and learning (Rapaport and Brown 2008; Sato et al. 2012; Silk 2007; Tardif 1994; Whiten and van de Waal 2018). This approach to infanticide includes acts derived from social pathologies and lack of experience (i.e., with no apparent benefit) and/or situations wherein an individual obtains any benefit, either direct or indirect, from the victim's death (Hausfater and Hrdy 1984; Hrdy 1979).

We performed a comprehensive literature review spanning 54 years (1970–2024), using the online databases SciELO, Scopus, and Web of Science. Studies in English, French, Portuguese, and Spanish were found by searching for the following combination of words and the respective translations: [“male/female” AND “infanticide” AND “primate*”]; [“male/female” AND “abuse” AND “primate*”]; [“male/female” AND “neglect” AND “primate*”]; [“male/female” AND “kidnapping” AND “primate*”]; [“male/female” AND “aunting to death” AND “primate*”]. It is important to note that we employed the keyword “primate*” to constrain our search to the focal taxonomic order. Although this decision likely omitted studies that did not explicitly use the term, the resulting dataset still provides a robust and unbiased representation of sex‐specific patterns of infanticide across primates.

Our research included the following sources: (i) book chapters that directly address the topic; (ii) original and published articles; (iii) datasets; (iv) published studies fully available in electronic format. We excluded the following research: (i) duplicated publications; (ii) preprints; (iii) studies that do not directly address the topic; and (iv) references considered grey literature (non‐peer‐reviewed literature, symposium summaries, notes, theses, and dissertations). To avoid double‐counting, we extracted data only from primary sources. For review articles, we used their reference lists solely to locate the corresponding primary studies and did not include the reviews themselves in our quantitative counts. This procedure ensured that each infanticide event in our database was counted only once. Data from studies that reported these terms in wild or captive primate populations were included. We confirmed the taxonomy of primate families and species using the website “Integrated Taxonomic Information System” of the Smithsonian Institution (https://www.itis.gov/) (Ann et al. 2004; Jäntschi and Sestraş 2011). The complete list of references used to obtain and analyze the data is available as Electronic supporting Material.

For the purpose of selecting studies that use the term “infanticide”, we considered those that describe cases of infanticide either by direct observation or inference (Kane and Gnépa 2016; Lowe et al. 2019; Mitani et al. 2012; Robbins et al. 2013). Infanticides reported by inference include fatal injuries observed on an infant's body, the disappearance of an infant after an attack, and/or an infant's permanent separation from the mother as the only way to survive (Lowe et al. 2019; Mitani et al. 2012; Robbins et al. 2013). When direct observation of the fatal act was unavailable, infanticide was inferred based on contextual and post‐event evidence reported in the original studies. These inferential cases included: (i) the disappearance of an infant following the immigration of an external male or changes in group composition (e.g., Nishie and Nakamura 2018; Robbins et al. 2014; Zipple et al. 2017); (ii) the subsequent death of an infant following episodes of intense aggression directed toward the infant or its mother (e.g., Alvarez et al. 2015; Kenyon et al. 2023) and (iii) infants found dead with injuries consistent with attacks by adult conspecifics during intergroup conflicts or generalized aggressive encounters (e.g., Harris and Monfort 2003; Knott et al. 2019; Robbins et al. 2014). These criteria are commonly used in primate studies when direct observation is not possible (Hrdy 1979; Lowe et al. 2019; Mitani et al. 2012; Robbins et al. 2013).

When researching the term “abuse”, we considered studies wherein the definition of this behavior consists of aggressive acts that resulted in the death of an infant by physical injury (biting, hitting, crushing, throwing, and trauma) (e.g., Brent et al. 2002; Maestripieri 2005; Maestripieri and Carroll 1998a, 2000). As for the term “neglect”, we selected studies that defined the term as the complete interruption of parental care culminating in the death of an infant from starvation (Leong et al. 2004), dehydration (Redman and Schneider 1979), or hypothermia (Kumamoto and Houck 2001).

Regarding “kidnapping”, this refers to cases in which a non‐maternal individual physically removes an infant from its mother and carries or retains it despite the mother's attempts to resist, until the infant dies as a direct consequence of the abduction (Shopland and Altmann 1987; Silk 1980). Death may result either from physical trauma inflicted by the kidnapper (Isbell et al. 2009; Mohnot 1980; Quiatt 1979) or from subsequent deprivation of maternal care (Hopf 1981; Paul and Kuester 1996). Regarding “aunting to death,” we included cases in which an inexperienced or younger female removed an infant from its mother and engaged in prolonged handling or carrying that resulted in the infant's death due to inadequate care or nourishment (Hrdy 1979; Hsu et al. 2000; Redman and Schneider 1979).

Regarding the behaviors labeled as “abuse”, “neglect”, “kidnapping”, and “aunting to death”, we used a formal distinction between direct and indirect infanticide in non‐human primates, following Hrdy (1979) and Hausfater and Hrdy (1984) framework. Direct infanticide refers to physical aggression toward infants, such as attacks, “abuse,” or “kidnapping” that causes injuries. Indirect infanticide involves infant deaths resulting from secondary effects, that is, through the lack of care or proper care (e.g., “fatal neglect,” “aunting to death,” or “kidnapping” without actual aggression). This distinction aims to clarify that, although males and females commit infanticide in similar numbers, they tend to employ different strategies.

Some studies lacked the essential information necessary for our analysis and, thus, were excluded. Specifically, we excluded studies that, despite mentioning the behaviors cited above, did not report fatal consequences (see Graves et al. 2002; Troisi and D'Amato 1991), as it would be impossible to determine whether these behaviors resulted in death. In addition, we did not include studies that, despite using the terms “infanticide”, “abuse”, “fatal neglect”, “kidnapping”, and “aunting to death”, did not specify the sex of the perpetrator, making it impossible to ascertain whether the perpetrator was male or female.

As a first step, we categorized the behaviors according to the terminology used by the original authors. Thus, we followed the authors' original classification when a study described a behavior using a specific label (e.g., “infanticide”, “abuse”, “neglect”, “kidnapping”, or “aunting to death”). For our comparative analyses, we later considered all these behaviors as forms of infanticide, following the approach of Hrdy (1979) and Hausfater and Hrdy (1984).

It is also important to note that we did not assess intentionality in the selection and classification of cases. Determining intent in non‐human animals is methodologically problematic, since behavioral outcomes may not reliably reflect underlying motivations (Burkart and van Schaik 2020; Kaufmann 2015; Rodrigues and Fröhlich 2024). Moreover, classic works such as Hrdy (1979) and Hausfater and Hrdy (1984) did not treat intentionality as a criterion for defining or categorizing infanticide, a precedent we follow here. Our inclusion of the previously cited behaviors is therefore based on their clear outcome, infant death, which ensures consistency across sources and maintains an objective focus. Furthermore, it is important to note that the expression of infanticide can vary markedly across primate lineages, often influenced by phylogenetically derived social systems. While this variation is valuable for understanding the diversity of primate societies, a detailed comparative analysis lies beyond the scope of this study and does not affect the conclusions drawn herein.

The data were analyzed using the chi‐square with Yates correction to compare the difference between sexes in studies that use the terms “infanticide”, “abuse”, “fatal neglect”, “kidnapping”, and “aunting to death” (R Core Team 2018). The RStudio program, version 4.0.5, package stats (R Core Team 2018) was employed for the statistical analysis. Statistical significance was attained when *p* ≤ 0.05 (R Core Team 2018).

## 3 Results

Our literature review of non‐human primate studies reporting infanticide events resulted in 238 references, consisting of 231 articles and seven book chapters (see Electronic supporting Material). These studies involved 87 different species distributed in 16 primate families (see Electronic supporting Material).

The analyses indicate that studies that specifically use the term “infanticide” report adult males (*N*_m_ = 135) committing this act significantly more often than adult females (*N*_f_ = 53) (*N* = 188, X² = 34.89, df = 1, *p* < 0.001). Regarding “abuse”, females and males exhibit an opposite result, that is, females (*N*_f_ = 30) were significantly more involved than males (*N*_m_ = 0) in abusive events (*N* = 30, X² = 28.03, df = 1, *p* < 0.001). Similarly, the term “neglect” is also used significantly more often when females are involved than when males are involved (*N*_m_ = 0, *N*_f_ = 42, *N* = 41, X² = 40.24, df = 1, *p* < 0.001). For “kidnapping”, all reported cases involved females (*N*_m_ = 1, *N*_f_ = 10, *N* = 11, X² = 7.36, df = 1, *p* < 0.01). For “aunting to death”, only females were implicated (*N*_m_ = 0, *N*_f_ = 2), and due to the small number of cases, no meaningful statistical analysis could be performed.

**Figure 1:**
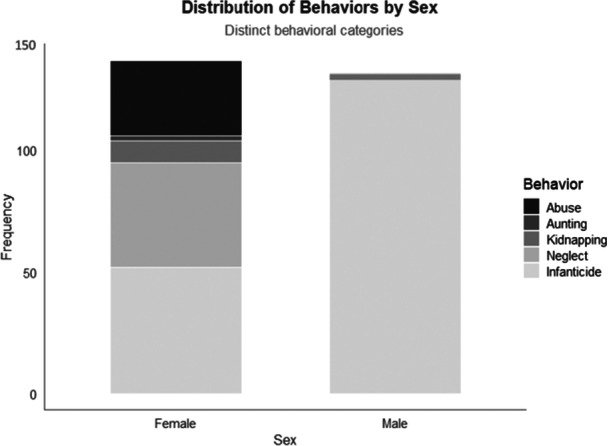
Number of events termed as “abuse”, “aunting”, “kidnapping”, “neglect” and “infanticide” in accordance with the sex of the perpetrators. See text for statistical results.

Adding together “infanticide,” “abuse,” “neglect,” “kidnapping,” and “aunting to death” to describe lethal actions toward infants, the difference between sexes becomes non‐significant (*N*_m_ = 131, *N*_f_ = 137, N = 268, df = 1, X² = 0.09, *p* = 0.76) (Figure 1).

## 4 Discussion

Our results point to two important findings involving an infant's death by non‐human primates: (i) the lethal behaviors labeled as “abuse”, “fatal neglect”, “kidnapping”, and “aunting to death” are more frequently attributed to females, whereas the term “infanticide” is more commonly attributed to males; and (ii) when “abuse”, “fatal neglect”, “kidnapping”, and “aunting to death” are properly classified as infanticide (Hausfater and Hrdy 1984; Hrdy 1979), sex differences become non‐significant. The second point aligns with our expectation that the observed differences between males and females in previous studies may have resulted from the inappropriate exclusion of behaviors classified as “abuse”, “fatal neglect”, “kidnapping”, and “aunting to death”.

In most studies, among the four behaviors, fatal “abuse” stands out as it always involves explicit physical aggression, representing a clear and direct form of infanticide (Hrdy 1979; Hrdy & Hausfater, 1984). However, our results point out that infanticide by female non‐human primates has been underreported since fatal abusive acts by this sex have often not been classified as infanticide in several studies (e.g., Brent et al. 2002; Maestripieri et al. 1999; Maestripieri and Carroll 1998b). In contrast, the term “infanticide” is almost always used, instead of “abuse”, when the aggressor is male (two exceptions: Rijksen 1981; Spijkerman et al. 1990). Notably, in human studies, “abuse” resulting in death is correctly classified as infanticide, irrespective of the perpetrator's sex (e.g., Arora et al. 2017; Ben Khelil et al. 2019; Chapdelaine‐Feliciati 2019; Costa and Victora 2021; East et al. 2022; Naviaux et al. 2020).

The other lethal behaviors (“kidnapping”, “fatal neglect”, and “aunting to death”) are generally less straightforward in terms of physical and direct aggression and would be classified as indirect infanticide (Hausfater and Hrdy 1984; Hrdy 1979). The exception here is “kidnapping” in specific cases when physical trauma is involved (e.g., Isbell et al. 2009; Mohnot 1980; Quiatt 1979). “Kidnapping” entails active interference and results in infant death, usually from deprivation of maternal care (e.g., Shopland and Altmann 1987; Silk 1980), whereas “fatal neglect” and “aunting to death” lead to infant death exclusively through inadequate care (e.g., “fatal neglect”: Maestripieri et al. 1997; Maestripieri and Carroll 2000; “Aunting to death”: Hrdy 1979; Hsu et al. 2000; Redman and Schneider 1979). It is possible that, because direct physical aggression is absent in most fatal cases involving these behaviors, they have been rarely treated as infanticide in non‐human primate literature (except: Shopland and Altmann 1987), despite aligning with the classical definition of infanticide. As demonstrated in our findings, these behaviors occur significantly more in females than in males. An exception is the study by Collins et al. (1984), which reported two fatal kidnappings by males, in which infants died from deprivation of maternal care. The distribution of these behaviors between sexes may reflect different aspects of primate social dynamics (Bercovitch 2002; Ebensperger 1998; Kokko and Jennions 2012; Quiatt 1979). For “fatal neglect” and “aunting to death”, the higher female involvement likely stems from the fact that nurturing and alloparental care in most primate species typically falls to females rather than males (Bercovitch 2002; Hrdy 1976; Kokko and Jennions 2012). Thus, while females are the main perpetrators when “fatal neglect” and “aunting to death” occur, this is not surprising. However, the question that arises is: why have clear cases of female‐perpetrated infanticide gone unrecorded as such in several studies?

One possible explanation involves a limited or inconsistent use of the term “infanticide” itself, which may have excluded indirect actions or less intense forms of aggression. Another possibility is that researchers may have simply considered such events unimportant or not worthy of classification as infanticide in their specific studies. However, this asymmetrical pattern suggests a more complex explanation involving both biological and cultural factors. From a biological perspective, the reason(s) why females exhibit indirect lethal behaviors (such as neglect) rather than overt aggression remains uncertain. One compelling explanation for the neglect cases involves the “baby schema” effect. This refers to a set of infantile features that biologically trigger caregiving responses and attenuate aggression in humans and possibly other animals (Lorenz 1943). Neuroimaging has provided neurophysiological evidence for its role in facilitating human nurturing behavior (Atzil et al. 2011; Glocker et al. 2009). Although a body of investigations in humans has confirmed its nurturing effect, particularly among females (e.g., Atzil et al. 2011; Glocker et al. 2009), systematic studies on other animals are still needed (Kawaguchi and Waller 2024). Despite this limitation, the efficacy of something similar to the “baby schema” effect in other primates is supported by documented cases of cross‐species adoption, where females have adopted unrelated infants of different species, indicating a response to infantile cues that can override species‐specific boundaries (e.g., Izar et al. 2006; Maestripieri 2001). In this view, caregiving mechanisms in other primates may also inhibit direct aggression while still allowing indirect harm through abandonment. Moreover, the nurturing response to infantile cues could also be behind some cases of “aunting to death” and “kidnapping”.

However, biological factors alone cannot fully explain the systematic underreporting of female‐perpetrated infanticide in previous studies, even if caregiving predispositions have contributed to reinforcing social expectations of female nurturing (Barnett 2013; Niall Hanlon 2012; Pullen and Vachhani 2021; Pun et al. 2004). In this regard, the literature stresses that in different societies, across different periods of time, the feminine archetype is linked to affection, empathy, and care (Barnett 2013; Niall Hanlon 2012; Pullen and Vachhani 2021; Pun et al. 2004; Sayer 2005; Umeh 1982). Nowadays, most contemporary human societies still tend to consider women as responsible for caring for children and other family members (Alesina et al. 2013; Charmes 2022; Pullen and Vachhani 2021; Sayer 2005). This assumption of a feminine archetype likely clouded researchers' perception, influencing them to label female‐perpetrated cases as “abuse”, “neglect”, or “kidnapping”, leaving the term “infanticide” unmentioned.

This is not the first time cultural factors have influenced scientific results. A particularly relevant example is how such influence affected George Murray Levick's discoveries about Adélie penguins (Guly 2016; Majdic 2021; Russell et al. 2012). When he began his observations, he was highly surprised by some behaviors exhibited by these penguins, such as males mating with other males, as well as with dead females (Majdic 2021; Rice et al. 2013; Russell et al. 2012), and he described these behaviors as “socially inappropriate” (Majdic 2021; Rice et al. 2013). When he completed his research, Levick considered publishing his results but was discouraged by his peers, who believed his findings were “too much for society” (Majdic 2021). After a century, the studies were rediscovered and published by the Natural History Museum of London (Majdic 2021; Russell et al. 2012). Thus, in a number of cases where females were involved, the term “infanticide” may have been omitted due to a similar culturally influenced bias.

Based on the considerations mentioned above, we gain deeper insights and precision into the infanticide phenomenon by adopting and being attentive to Hrdy (1979) and Hausfater and Hrdy (1984) definition. However, as some points of their framework are scattered, we propose a clarified compilation of their classic definition for use in future research: “Infanticide refers to direct or indirect behaviors that fatally harm an infant, from which the conspecific perpetrator may derive direct, indirect, or no benefit”. This compiled definition should minimize potential sex‐based biases in the study of infanticide, promoting a clear and accurate understanding of the phenomenon, describing distinct behavioral strategies or mechanisms through which infanticide is carried out, thereby adding specificity to how infant mortality occurs within this broader framework. By adopting the classical definition in its full scope, researchers gain a framework that facilitates a more comprehensive examination of infant mortality and reduces the systematic underreporting and misclassification of female‐perpetrated cases.

## Author Contributions

**Tatiani G Albert** conceived the research, collected the data, and wrote the manuscript. **Nicola Schiel** contributed to writing and reviewing the manuscript. **Marcelo A Ramos** contributed to writing and reviewing the manuscript. **Antonio Souto** conceived the research and wrote the manuscript.

## Ethics Statement

Ethical approval is not applicable to this study, as it is a review of published literature.

## Conflicts of Interest

The authors declare no conflicts of interest.

## Supporting information

Electronic Supplementary Material ‐ ‐ artigo termos.

## Data Availability

Complete datasets with information from the articles used in the analysis can be found in the Electronic Supporting Material.

## References

[ajp70132-bib-0002] Alesina, A. , P. Giuliano , and N. Nunn . 2013. “On the Origins of Gender Roles: Women and the Plough.” Quarterly Journal of Economics 128, no. 2: 469–530. 10.1093/qje/qjt005.

[ajp70132-bib-0003] Alvarez, S. , A. Di Fiore , J. Champion , M. S. Pavelka , J. Páez , and A. Link . 2015. “Male‐Directed Infanticide in Spider Monkeys (Ateles spp).” Primates 56, no. 2: 173–181. 10.1007/s10329-014-0454-y. 25373339

[ajp70132-bib-0004] Ann, C. , S. Vertebrate , Z. Librarianisenior , and R. Librarian . 2004ITIS (The Integrated Taxonomic Information System).

[ajp70132-bib-0087] Arora, A. , J. Yadav , S. Yadav , and H. Singh . 2017. “Infanticide: A Concept.” Journal of Forensic Science and Medicine 3, no. 1: 42. 10.4103/jfsm.jfsm_51_15.

[ajp70132-bib-0005] Atzil, S. , T. Hendler , and R. Feldman . 2011. “Specifying the Neurobiological Basis of Human Attachment: Brain, Hormones, and Behavior in Synchronous and Intrusive Mothers.” Neuropsychopharmacology 36, no. 13: 2603–2615. 10.1038/npp.2011.172. 21881566 PMC3230500

[ajp70132-bib-0006] Barnett, B. 2013. “Toward Authenticity: Using Feminist Theory to Construct Journalistic Narratives of Maternal Violence.” Feminist Media Studies 13, no. 3: 505–524. 10.1080/14680777.2012.708514.

[ajp70132-bib-0088] Ben Khelil, M. , I. Boukthir , O. Hmandi , M. Zhioua , and M. Hamdoun . 2019. “Trends of Infanticides in Northern Tunisia: A 40 years study (1977–2016).” Child Abuse and Neglect 95. 10.1016/j.chiabu.2019.104047. 31288130

[ajp70132-bib-0007] Bercovitch, F. B. 2002. “Sex‐Biased Parental Investment in Primates.” International Journal of Primatology 23, no. 4: 905–921.

[ajp70132-bib-0008] Bezerra, B. M. , A. D. S. Souto , and N. Schiel . 2007. “Infanticide and Cannibalism in a Free‐Ranging Plurally Breeding Group of Common Marmosets (*Callithrix jacchus*).” American Journal of Primatology 69, no. 8: 945–952. 10.1002/ajp.20394. 17253615

[ajp70132-bib-0010] Brent, L. , T. Koban , and S. Ramirez . 2002. “Abnormal, Abusive, and Stress‐Related Behaviors in Baboon Mothers.” Biological Psychiatry 52, no. 11: 1047–1056. 10.1016/S0006-3223(02)01540-8. 12460688

[ajp70132-bib-0011] Burkart, J. M. , and C. P. van Schaik . 2020. “Marmoset Prosociality Is Intentional.” Animal Cognition 23, no. 3: 581–594. 10.1007/s10071-020-01363-6. 32107657 PMC7181450

[ajp70132-bib-0089] Chapdelaine‐Feliciati, C. 2019. “Feminicides of Girl Children in the Family Context: An International Human Rights Law Approach.” Brill Research Perspectives in Family Law in a Global Society 1, no. 3: 1–81. 10.1163/24058386-12340003.

[ajp70132-bib-0013] Charmes, J. 2022. “Variety and Change of Patterns in the Gender Balance Between Unpaid Care‐Work, Paid Work and Free Time Across the World and Over Time: A Measure of Wellbeing?” Wellbeing, Space and Society 3: 100081. 10.1016/j.wss.2022.100081.

[ajp70132-bib-0090] Collins, D. A. , C. D. Busse , and J. Goodall . 1984. “Infanticide in Two Populations of Savanna Baboons.” In Infanticide: Comparative and Evolutionary Perspectives, edited by E. G. Hausfater and S. B. Hrdy , 10th ed., 193–216. 10.4324/9780203788608.

[ajp70132-bib-0084] Costa, J. C. , and C. G. Victora . 2021. “A Scoping Review of Methods for Assessment of sex Differentials in Early Childhood Mortality.” BMC Pediatrics 21, no. 1. 10.1186/s12887-021-02503-8. PMC7836200 33499809

[ajp70132-bib-0014] Culot, L. , Y. Lledo‐Ferrer , O. Hoelscher , F. J. J. Muñoz Lazo , M.‐C. Huynen , and E. W. Heymann . 2011. “Reproductive Failure, Possible Maternal Infanticide, and Cannibalism in Wild Moustached Tamarins, *Saguinus mystax* .” Primates 52, no. 2: 179–186. 10.1007/s10329-011-0238-6. 21328068 PMC3068257

[ajp70132-bib-0015] Digby, L. 2009. Infanticide by Female Mammals: Implications for the Evolution of Social Systems. Infanticide by Males and Its Implications 423–446. 10.1017/cbo9780511542312.019.

[ajp70132-bib-0085] East, M. L. , D. Thierer , S. Benhaiem , S. Metzger , and H. Hofer . 2022. “Infanticide by Adult Females Causes Sexual Conflict in a Female‐Dominated Social Mammal.” Frontiers in Ecology and Evolution 10: 860854. 10.3389/fevo.2022.860854.

[ajp70132-bib-0017] Ebensperger, L. A. 1998. “Strategies and Counterstrategies to Infanticide in Mammals.” Biological Reviews 73: 1.

[ajp70132-bib-0018] Glocker, M. L. , D. D. Langleben , K. Ruparel , J. W. Loughead , R. C. Gur , and N. Sachser . 2009. “Baby Schema in Infant Faces Induces Cuteness Perception and Motivation for Caretaking in Adults.” Ethology 115, no. 3: 257–263. 10.1111/j.1439-0310.2008.01603.x. 22267884 PMC3260535

[ajp70132-bib-0083] Graves, F. C. , K. Wallen , and D. Maestripieri . 2002. “Opioids and Attachment in Rhesus Macaque (Macaca mulatta) Abusive Mothers.” Behavioral Neuroscience 116, no. 3: 489–493. 10.1037/0735-7044.116.3.489. 12049330

[ajp70132-bib-0019] Guly, H. R. 2016. “George Murray Levick. 1876–1956, Antarctic Explorer.” Journal of Medical Biography 24, no. 1: 4–10. 10.1177/0967772014533051. 24974150

[ajp70132-bib-0020] Hanlon, N. 2012. Masculinities and Care. In Victoria Robinson & Diane Richardson (Eds.), Masculinities, Care and Equality: Identity and Nurture in Men's Lives (Vol. 1st, pp. 43–65). Palgrave Macmillan.

[ajp70132-bib-0021] Harris, T. R. , and S. L. Monfort . 2003. “Behavioral and Endocrine Dynamics Associated With Infanticide in a Black and White Colobus Monkey (*Colobus guereza*).” American Journal of Primatology 61, no. 3: 135–142. 10.1002/ajp.10116. 14610731

[ajp70132-bib-0022] Hausfater, G. , and S. B. Hrdy . 1984. Infanticide: Comparative and Evolutionary Perspectives (1st ed.). Routledge. 10.4324/9780203788608.

[ajp70132-bib-0023] Hopf, S. 1981. “Conditions of Failure and Recovery of Maternal Behavior in Captive Squirrel Monkeys (Saimiri).” International Journal of Primatology 2, no. 4: 335–349. 10.1007/BF02693483.

[ajp70132-bib-0024] Hrdy, S. B. 1976. “Care and Exploitation of Nonhuman Primate Infants by Conspecifics Other Than the Mother.” Advances in the Study of Behavior 6, no. C: 101–158. 10.1016/S0065-3454(08)60083-2.

[ajp70132-bib-0025] Hrdy, S. B. 1979. “Infanticide Among Animals: A Review, Classification, and Examination of the Implications for the Reproductive Strategies of Females.” Ethology and Sociobiology 1, no. 1: 13–40. 10.1016/0162-3095(79)90004-9.

[ajp70132-bib-0026] Hsu, M. J. , J.‐F. Lin , and G. Agoramoorthy . 2000. “Occurrence of Twins in Wild Formosan Macaques, *Macaca cyclopis*, at Mt. Longevity, Taiwan.” Folia Primatologica 71, no. 3: 154–156. 10.1159/000021745. 10828694

[ajp70132-bib-0028] Isbell, L. A. , T. P. Young , K. E. Jaffe , A. A. Carlson , and R. L. Chancellor . 2009. “Demography and Life Histories of Sympatric Patas Monkeys, *Erythrocebus patas*, and Vervets, *Cercopithecus aethiops*, in Laikipia, Kenya.” International Journal of Primatology 30, no. 1: 103–124. 10.1007/s10764-009-9332-7. PMC2949556 20976285

[ajp70132-bib-0029] Izar, P. , M. P. Verderane , E. Visalberghi , et al. 2006. “Cross‐Genus Adoption of a Marmoset (*Callithrix jacchus*) by Wild Capuchin Monkeys (*Cebus libidinosus*): Case Report.” American Journal of Primatology 68, no. 7: 692–700. 10.1002/ajp.20259. 16786521

[ajp70132-bib-0030] Jäntschi, L. , and R. E. Sestraş . 2011. “Local Using of Integrated Taxonomic Information System (ITIS).” Bulletin UASVM Horticulture 68, no. 1: 1. http://www.itis.gov/downloads/.

[ajp70132-bib-0032] Kane, E. , and F. Gnépa . 2016. “An Infanticide Attempt After Male Takeover in Diana Monkeys (*Cercopithecus diana diana*) in Taï, Côte d'Ivoire.” African Primates 11, no. 1: 37–40.

[ajp70132-bib-0033] Kaufmann, A. 2015. “Animal Mental Action: Planning Among Chimpanzees.” Review of Philosophy and Psychology 6, no. 4: 745–760. 10.1007/s13164-014-0228-x.

[ajp70132-bib-0034] Kawaguchi, Y. , and B. M. Waller . 2024. “Lorenz's Classic 'Baby Schema': A Useful Biological Concept?” Proceedings of the Royal Society B: Biological Sciences 291, no. 2025: 20240570. 10.1098/rspb.2024.0570. PMC11285920 38889779

[ajp70132-bib-0035] Kenyon, M. , N. T. Phoung , V. T. Binh , and A. Cronin , Org . (2023). The Development of Care Protocols for Pygmy Lorises (*Xanthonycticebus pygmaeus*) From 2008 to 2023 at the Dao Tien Endangered Primate Species Centre, Cat Tien National Park, Vietnam. In Vietnamese Journal of Primatology (Vol. 3, Issue 4).

[ajp70132-bib-0036] Knott, C. D. , A. M. Scott , C. A. O'Connell , et al. 2019. “Possible Male Infanticide in Wild Orangutans and a Re‐Evaluation of Infanticide Risk.” Scientific Reports 9, no. 1: 7806. 10.1038/s41598-019-42856-w. 31127126 PMC6534599

[ajp70132-bib-0037] Kokko, H. , and M. D. Jennions . 2012. Sex Differences in Parental Care (Nick J. Royle, Per T. Smiseth, & Mathias Kölliker, Eds.; pp. 101–116).

[ajp70132-bib-0038] Kumamoto, A. T. , and M. L. Houck . 2001. “Cytogenetic Identification of a Hybrid Owl Monkey, *Aotus lemurinus griseimembra* .” Journal of Zoo and Wildlife Medicine 32, no. 1: 130–133. 10.1638/1042-7260(2001)032. 12790410

[ajp70132-bib-0039] Leong, K. M. , S. P. Terrell , and A. Savage . 2004. “Causes of Mortality in Captive Cotton‐Top Tamarins (*Saguinus oedipus*).” Zoo Biology 23, no. 2: 127–137. 10.1002/zoo.10121.

[ajp70132-bib-0040] Lorenz, K. 1943. “Die Angeborenen Formen Möglicher Erfahrung.” Zeitschrift für Tierpsychologie 5, no. 2: 235–409. 10.1111/j.1439-0310.1943.tb00655.x.

[ajp70132-bib-0041] Lowe, A. E. , C. Hobaiter , C. Asiimwe , K. Zuberbühler , and N. E. Newton‐Fisher . 2019. “Intra‐Community Infanticide in Wild, Eastern Chimpanzees: A 24‐year Review.” Primates 61, no. 1: 69–82. 10.1007/s10329-019-00730-3. 31134473 PMC6971177

[ajp70132-bib-0042] Lukas, D. , and E. Huchard . 2014. “The Evolution of Infanticide by Males in Mammalian Societies.” Science 346, no. 6211: 841–844. 10.1126/science.1257226. 25395534

[ajp70132-bib-0043] Lukas, D. , and E. Huchard . 2019. “The Evolution of Infanticide by Females in Mammals.” Philosophical Transactions of the Royal Society, B: Biological Sciences 374, no. 1780: 20180075. 10.1098/rstb.2018.0075. PMC6664130 31303157

[ajp70132-bib-0044] Maestripieri, D. 2001. “Is There Mother‐Infant Bonding in Primates?” Developmental Review 21, no. 1: 93–120. 10.1006/drev.2000.0522.

[ajp70132-bib-0045] Maestripieri, D. 2005. “Early Experience Affects the Intergenerational Transmission of Infant Abuse in Rhesus Monkeys.” Proceedings of the National Academy of Sciences 102, no. 27: 9726–9729. 10.1073/pnas.0504122102. PMC1172276 15983367

[ajp70132-bib-0046] Maestripieri, D. , and K. A. Carroll . 1998a. “Behavioral and Environmental Correlates of Infant Abuse in Group‐Living Pigtail Macaques.” Infant Behavior and Development 21, no. 4: 603–612. 10.1016/S0163-6383(98)90032-7.

[ajp70132-bib-0047] Maestripieri, D. , and K. A. Carroll . 1998b. “Risk Factors for Infant Abuse and Neglect in Group‐Living Rhesus Monkeys.” Psychological Science 9, no. 2: 143–145. 10.1111/1467-9280.00027.

[ajp70132-bib-0048] Maestripieri, D. , and K. A. Carroll . 2000. “Causes and Consequences of Infant Abuse and Neglect in Monkeys.” Aggression and Violent Behavior 5, no. 3: 245–254. 10.1016/S1359-1789(98)00019-6.

[ajp70132-bib-0049] Maestripieri, D. , M. Tomaszycki , and K. A. Carroll . 1999. “Consistency and Change in the Behavior of Rhesus Macaque Abusive Mothers With Successive Infants.” Developmental Psychobiology 34, no. 1: 29–35. 10.1002/(SICI)1098-2302(199901)34:1<29::AID-DEV5>3.0.CO;2-U. 9919431

[ajp70132-bib-0091] Maestripieri, D. , K. Wallen , and K. A. Carroll . 1997. “Genealogical and Demographic Influences on Infant Abuse and Neglect in Group‐Living Sooty Mangabeys (*Cercocebus atys*).” Developmental Psychobiology 31, no. 3: 175–180. 10.1002/(SICI)1098-2302(199711)31:3<175::AID-DEV2>3.0.CO;2-P. 9386919

[ajp70132-bib-0050] Majdic, G. 2021. Chapter 15: Depraved Hooligan Penguins. In Soul Mate Biology (pp. 113–120). Springer International Publishing. 10.1007/978-3-030-67212-6.

[ajp70132-bib-0052] Mitani, J. C. , J. Call , P. M. Kappeler , R. A. Palombit , and J. B. Silk . 2012. “Infanticide: Male Strategies and Female Counterstrategies.” In The evolution of primate societies, 432–468. University of Chicago Press.

[ajp70132-bib-0053] Mohnot, S. M. 1980. “Intergroup Infant Kidnapping in Hanuman Langur.” Folia Primatologica 34, no. 3–4: 259–277. 10.1159/000155958. 7216004

[ajp70132-bib-0086] Naviaux, A.‐F. , P. Janne , and M. Gourdin . 2020. “Psychiatric Considerations on Infanticide: Throwing the Baby out With the Bathwater.” Psychiatria Danubina 32: 24–28. 32890357

[ajp70132-bib-0054] Nishie, H. , and M. Nakamura . 2018. “A Newborn Infant Chimpanzee Snatched and Cannibalized Immediately After Birth: Implications for ‘Maternity Leave’ in Wild Chimpanzee.” American Journal of Physical Anthropology 165, no. 1: 194–199. 10.1002/ajpa.23327. 28983906

[ajp70132-bib-0056] Palombit, R. A. 2015. “Infanticide as Sexual Conflict: Coevolution of Male Strategies and Female Counterstrategies.” Cold Spring Harbor Perspectives in Biology 7, no. 6: a017640. 10.1101/cshperspect.a017640. 25986557 PMC4448612

[ajp70132-bib-0057] Paul, A. , and J. Kuester . 1996. “Infant Handling by Female Barbary Macaques (*Macaca sylvanus*) at Affenberg Salem: Testing Functional and Evolutionary Hypotheses.” Behavioral Ecology and Sociobiology 39, no. 2: 133–145. 10.1007/s002650050275.

[ajp70132-bib-0058] Pullen, A. , and S. J. Vachhani . 2021. “Feminist Ethics and Women Leaders: From Difference to Intercorporeality.” Journal of Business Ethics 173, no. 2: 233–243. 10.1007/s10551-020-04526-0. PMC7214853 32398886

[ajp70132-bib-0059] Pun, S. H. , J. L. C. Ma , and K. C. C. Lai . 2004. “In Search of Perfect Motherhood for Imperfect Childhood—Experiences of 22 Chinese Mothers.” Child & Family Social Work 9, no. 3: 285–293. 10.1111/j.1365-2206.2004.00311.x.

[ajp70132-bib-0060] Quiatt, D. 1979. “Aunts and Mothers: Adaptive Implications of Allomaternal Behavior of Nonhuman Primates.” American Anthropologist 81, no. 2: 310–319. 10.1525/aa.1979.81.2.02a00040.

[ajp70132-bib-0061] R Core Team . 2018. R: A Language and Environment for Statistical Computing. In R Core Team. Foundation for Statistical Computing.

[ajp70132-bib-0062] Rapaport, L. G. , and G. R. Brown . 2008. “Social Influences on Foraging Behavior in Young Nonhuman Primates: Learning What, Where, and How to Eat.” Evolutionary Anthropology: Issues, News, and Reviews 17, no. 4: 189–201. 10.1002/evan.20180.

[ajp70132-bib-0063] Redman, H. C. , and R. Schneider . 1979. “An Epidemiological Study of Neonatal and Postneonatal Mortality in Macaco radiata at the California Primate Research Center (1966–1973).” Journal of Medical Primatology 8, no. 1: 1–17. 10.1159/000460171. 113534

[ajp70132-bib-0064] Rice, W. R. , U. Friberg , and S. Gavrilets . 2013. “Homosexuality via Canalized Sexual Development: A Testing Protocol for a New Epigenetic Model.” BioEssays 35, no. 9: 764–770. 10.1002/bies.201300033. 23868698 PMC3840696

[ajp70132-bib-0065] Rijksen, H. D. 1981. “Infant Killing: A Possible Consequence of a Disputed Leader Role.” Behaviour 78, no. 1: 138–168. 10.1163/156853981x00293.

[ajp70132-bib-0066] Robbins, A. M. , M. Gray , A. Basabose , et al. 2013. “Impact of Male Infanticide on the Social Structure of Mountain Gorillas.” PLoS One 8, no. 11: e78256. 10.1371/journal.pone.0078256. 24223143 PMC3819382

[ajp70132-bib-0067] Robbins, A. M. , M. Gray , P. Uwingeli , I. Mburanumwe , E. Kagoda , and M. M. Robbins . 2014. “Variance in the Reproductive Success of Dominant Male Mountain Gorillas.” Primates 55, no. 4: 489–499. 10.1007/s10329-014-0426-2. 24818867

[ajp70132-bib-0068] Rodrigues, E. D. , and M. Fröhlich . 2024. “Operationalizing Intentionality in Primate Communication: Social and Ecological Considerations.” International Journal of Primatology 45, no. 3: 501–525. 10.1007/s10764-021-00248-w.

[ajp70132-bib-0069] Russell, D. G. D. , W. J. L. Sladen , and D. G. Ainley . 2012. “Dr. George Murray Levick (1876–1956): Unpublished Notes on the Sexual Habits of the Adélie Penguin.” Polar Record 48, no. 4: 387–393. 10.1017/S0032247412000216.

[ajp70132-bib-0070] Sato, A. , H. Koda , A. Lemasson , S. Nagumo , and N. Masataka . 2012. “Visual Recognition of Age Class and Preference for Infantile Features: Implications for Species‐Specific vs Universal Cognitive Traits in Primates.” PLoS One 7, no. 5: e38387. 10.1371/journal.pone.0038387. 22685529 PMC3368701

[ajp70132-bib-0071] Sayer, L. C. 2005. Gender, Time and Inequality: Trends in Women's and Men's Paid Work, Unpaid Work and Free Time Downloaded from. In Social Forces (Vol. 84, Issue 1). http://sf.oxfordjournals.org/.

[ajp70132-bib-0073] Shopland, J. M. , and J. Altmann . 1987. “Fatal Intragroup Kidnapping in Yellow Baboons.” American Journal of Primatology 13, no. 1: 61–65. 10.1002/ajp.1350130108. 31973488

[ajp70132-bib-0074] Silk, J. B. 1980. “Kidnapping and Female Competition Among Captive Bonnet Macaques.” Primates 21, no. 1: 100–110. 10.1007/BF02383827.

[ajp70132-bib-0075] Silk, J. B. 2007. Social Components of Fitness in Primate Groups. In Science (Vol. 317, Issue 5843, pp. 1347–1351). 10.1126/science.1140734. 17823344

[ajp70132-bib-0076] Spijkerman, R. P. , J. A. R. A. M. Van Hooff , and W. Jens . 1990. “A Case of Lethal Infant Abuse in an Established Group of Chimpanzees.” Folia Primatologica 55, no. 1: 41–44. 10.1159/000156496. 2394415

[ajp70132-bib-0078] Tardif, S. D. 1994. “Relative Energetic Cost of Infant Care in Small‐Bodied Neotropical Primates and Its Relation to Infant‐Care Patterns.” American Journal of Primatology 34, no. 2: 133–143. 10.1002/ajp.1350340205. 31936968

[ajp70132-bib-0082] Troisi, A. , and F. R. D'Amato . 1991. “Anxiety in the Pathogenesis of Primate Infant Abuse: A Pharmacological Study.” Psychopharmacology 103, no. 4: 571–572. 10.1007/BF02244261. 1676532

[ajp70132-bib-0079] Umeh, M. A. 1982. “The Joys of Motherhood: Myth or Reality?” Colby Quarterly 18, no. 1: 5.

[ajp70132-bib-0080] Whiten, A. , and E. van de Waal . 2018. The Pervasive Role of Social Learning in pRimate Lifetime Development. In Behavioral Ecology and Sociobiology (Vol. 72, Issue 5). Springer Verlag. 10.1007/s00265-018-2489-3. PMC5934467 29755181

[ajp70132-bib-0081] Zipple, M. N. , J. H. Grady , J. B. Gordon , et al. 2017. “Conditional Fetal and Infant Killing by Male Baboons.” Proceedings of the Royal Society B: Biological Sciences 284, no. 1847: 20162561. 10.1098/rspb.2016.2561. PMC5310045 28100822

