# Supplementary material for: Revisiting Infanticide in Non‐Human Primates Reveals a Similar Likelihood of Male and Female Perpetrators
Source: Am J Primatol. 2026 Mar 23;88(3):e70132. doi: 10.1002/ajp.70132 (PMC13006818; doi:10.1002/ajp.70132)
Supplement: Supplementary file 1 — Electronic Supplementary Material ‐ ‐ artigo termos. [file AJP-88-e70132-s001.docx]

# SUPPLEMENTARY DATA

**List of nonhuman primate studies reporting harmful behaviors that resulted in infant death.**

**SUPPLEMENTARY TABLE**

Primate families and species involved in studies reporting harmful behaviors that resulted in infant death, along with the original sources in which these events were documented. The numbers in the “Source Code” column correspond to the references listed at the end of the table.

| Family | **Species** | Source, Code |
| --- | --- | --- |
| Aotidae | *Aotus nancymaae* | [1] |
| Atelidae | *Alouatta caraya* | [2–4] |
|  | *Alouatta guariba* | [4,5] |
|  | *Alouatta palliata* | [6,7] |
|  | *Alouatta pigra* | [4,8–10] |
|  | *Alouatta seniculus* | [4,11] |
|  | *Ateles belzebuth* | [12,13] |
|  | *Ateles geoffroyi* | [12,13] |
|  | *Ateles paniscus* | [14] |
| Callitrichidae | *Callithrix flaviceps* | [15,16] |
|  | *Callithrix jacchus* | [17–30] |
|  | *Leontopithecus chrysomelas* | [31] |
|  | *Leontocebus fuscicollis* | [32] |
|  | *Oedipomidas oedipus* | [33–39] |
|  | *Saguinus geoffroyi* | [40] |
|  | *Saguinus mystax* | [41] |
| Cebidae | *Cebus apella* | [42,43] |
|  | *Cebus capucinus* | [44–49] |
|  | *Cebus olivaceus* | [50] |
|  | *Saimiri collinsi* | [51] |
|  | *Sairniri sciureus* | [52,53] |
|  | *Sapajus nigritus* | [54] |
| Cercopithecidae | *Cercocebus atys* | [48,55–59] |
|  | *Cercopithecus aethiops* | [60–62] |
|  | *Cercopithecus ascanius* | [63,64] |
|  | *Cercopithecus diana* | [65] |
|  | *Cercopithecus mitis* | [66–69] |
|  | *Cercopithecus lowei* | [52] |
|  | *Chlorocebus pygerythrus* | [60,70–72] |
|  | *Colobus guereza* | [73,74] |
|  | *Colobus vellerosus* | [75–77] |
|  | *Erythrocebus patas* | [61,78] |
|  | *Lophocebus albigena* | [79] |
|  | *Macaca arctoides* | [80] |
|  | *Macaca fascicularis* | [52,63,81–87] |
|  | *Macaca fuscata* | [72,88–94] |
|  | *Macaca mulatta* | [52,63,72,88,95–106] |
|  | *Macaca nemestrina* | [107–110] |
|  | *Macaca radiata* | [111,112] |
|  | *Macaca sylvanus* | [63,113,114] |
|  | *Macaca thibetana* | [115] |
|  | *Macaca tonkeana* | [116] |
|  | *Nasalis larvatus* | [117] |
|  | *Papio anubis* | [118–120] |
|  | *Papio cynocephalus* | [118,119,121–123] |
|  | *Papio hamadryas* | [63,124–136] |
|  | *Papio ursinus* | [63,118,119,137–142] |
|  | *Piliocolobus badius* | [143] |
|  | *Piliocolobus rufomitratus* | [144,145] |
|  | *Rhinopithecus bieti* | [146] |
|  | *Rhinopithecus roxellana* | [147–149] |
|  | *Semnopithecus entellus* | [52,63,88,150–155] |
|  | *Semnopithecus johnii* | [156] |
|  | *Semnopithecus vetulus* | [63,157] |
|  | *Theropithecus gelada* | [63,158–162] |
|  | *Trachypithecus cristatus* | [163] |
|  | *Trachypithecus francoisi* | [164] |
|  | *Trachypithecus leucocephalus* | [165] |
| Cheirogaleidae | *Mirza coquereli* | [166] |
| Daubentoniidae | *Daubentonia madagascariensis* | [167] |
| Galagidae | *Otolemur crassicaudatus* | [168] |
|  | *Galago senegalensis* | [168,169] |
|  | *Otolemur garnettii* | [169] |
| Hominidae | *Gorilla beringei* | [170] |
|  | *Gorilla gorilla* | [52,63,96,171–177] |
|  | *Pan troglodytes* | [63,178–212] |
|  | *Pongo pygmaeus* | [213,214] |
| Hylobatidae | *Hoolock hoolock* | [215] |
|  | *Hylobates lar* | [216] |
|  | *Nomascus concolor* | [217] |
| Indriidae | *Propithecus verreauxi* | [218,219] |
|  | *Propithecus edwardsi* | [220–222] |
|  | *Propithecus coquereli* | [223] |
| Lemuridae | *Eulemur fulvus* | [224] |
|  | *Eulemur macaco* | [168,224,225] |
|  | *Eulemur rufus* | [224] |
|  | *Lemur catta* | [224,226–231] |
|  | *Varecia variegata* | [168] |
| Lepilemuridae | *Lepilemur edwardsi* | [232] |
| Lorisidae | *Nycticebus javanicus* | [233] |
|  | *Perodicticus potto* | [168,234] |
|  | *Loris tardigradus* | [234] |
|  | *Xanthonycticebus pygmaeus* | [235] |
| Pitheciidae | *Callicebus nigrifrons* | [236] |
|  | *Pithecia pithecia* | [133] |
| Tarsiidae | *Cephalopachus bancanus* | [237] |
|  | *Tarsius tarsier* | [238] |

**Sources:**

1. Kumamoto AT, Houck ML. 2001 Cytogenetic identification of a hybrid owl monkey, *Aotus nancymaae* X *Aotus lemurinus griseimembra*. *Journal of Zoo and Wildlife Medicine* 32, 130–133. (doi:10.1638/1042-7260(2001)032)

2. Calegaro-Marques C, Bicca-Marques JC. 1996 Emigration in a black howling monkey group. *Int J Primatol* 17, 229–237. (doi:10.1007/BF02735450)

3. Pavé R, Kowalewski MM, Garber PA, Zunino GE, Fernandez VA, Peker SM. 2012 Infant Mortality in Black-and-Gold Howlers (*Alouatta caraya*) Living in a Flooded Forest in Northeastern Argentina. *Int J Primatol* 33, 937–957. (doi:10.1007/s10764-012-9626-z)

4. Crockett CM. 2003 Re-evaluating the sexual selection hypothesis for infanticide by Alouatta males. *Sexual Selection and Reproductive Competition in Primates: New Perspectives and Directions* , 327–365.

5. Martins V, Chaves ÓM, Neves MB, Bicca-Marques JC. 2015 Parturition and potential infanticide in free-ranging *Alouatta guariba clamitans*. *Primates* 56, 119–125. (doi:10.1007/s10329-015-0461-7)

6. Clarke MR. 1983 Infant-Killing and Infant Disappearance Following Male Takeovers in a Group of Free-Ranging Howling Monkeys (*Alouatta palliata*) in Costa Rica. *Am J Primatol* 5, 241–247. (doi:10.1002/ajp.1350050308)

7. Clarke MR, Zucker EL, Glander KE. 1994 Group takeover by a natal male howling monkey (*Alouatta palliata*) and associated disappearance and injuries of immatures. *Primates* 35, 435–442. (doi:10.1007/BF02381952)

8. van Belle S, Kulp AE, Thiessen-Bock R, Garcia M, Estrada A. 2010 Observed infanticides following a male immigration event in black howler monkeys, *Alouatta pigra*, at Palenque National Park, Mexico. *Primates* 51, 279–284. (doi:10.1007/s10329-010-0207-5)

9. Knopff KH, Knopff ARA, Pavelka MSM. 2004 Observed case of infanticide committed by a resident male central American black howler monkey (*Alouatta pigra*). *Am J Primatol* 63, 239–244. (doi:10.1002/ajp.20053)

10. García-Feria LM, Pérez BAC, Sánchez-Girón X. 2016 Nuevo registro de infanticidio en *Alouatta pigra*: Oportunidad de traición del altruismo. *Therya* 7, 193–196. (doi:10.12933/therya-16-328)

11. Agoramoorthy G, Rudran R. 1995 Infanticide by Adult and Subadult Males in Free‐ranging Red Howler Monkeys, *Alouatta seniculus*, in Venezuela. *Ethology* 99, 75–88. (doi:10.1111/j.1439-0310.1995.tb01090.x)

12. Gibson KN, Vick LG, Palma AC, Carrasco FM, Taub D, Ramos-Fernandez G. 2008 Intra-community infanticide and forced copulation in spider monkeys: A multi-site comparison between Cocha Cashu, Peru and Punta Laguna, Mexico. *Am J Primatol* 70, 485–489. (doi:10.1002/ajp.20511)

13. Alvarez S, Di Fiore A, Champion J, Pavelka MS, Páez J, Link A. 2015 Male-directed infanticide in spider monkeys (Ateles spp.). *Primates* 56, 173–181. (doi:10.1007/s10329-014-0454-y)

14. Shimooka Y, Campbell CJ, Di Fiore A, Felton AM, Izawa K, Link A, Nishimura A, Ramos-Fernández G, Wallace RB. 2008 Demography and group composition of Ateles. In *Spider monkeys: Behavior, ecology and evolution of the genus Ateles*, pp. 329–348. (doi:10.1017/cbo9780511721915.012)

15. Hilário RR, Ferrari SF. 2010 Double infanticide in a free-ranging group of buffy-headed marmosets, *Callithrix flaviceps*. *J Ethol* 28, 195–199. (doi:10.1007/s10164-009-0182-8)

16. Hilário RR, Ferrari SF. 2010 Four breeding females in a free-ranging group of buffy-headed marmosets (*Callithrix flaviceps*). *Folia Primatologica* 81, 31–40. (doi:10.1159/000278839)

17. Alonso C. 1986 Fracasso na inibição da reprodução de uma fêmea subordinada e troca de hierarquia em um grupo familiar de *Callithrix jacchus jacchus*. *A Primatologia No Brasil* 2, 202.

18. Arruda MF, Araújo A, Sousa MBC, Albuquerque FS, Albuquerque ACSR, Yamamoto ME. 2005 Two breeding females within free-living groups may not always indicate polygyny: Alternative subordinate female strategies in common marmosets (*Callithrix jacchus*). *Folia Primatologica* 76, 10–20. (doi:10.1159/000082451)

19. Bezerra BM, Souto ADS, Schiel N. 2007 Infanticide and cannibalism in a free-ranging plurally breeding group of common marmosets (*Callithrix Jacchus*). *Am J Primatol* 69, 945–952. (doi:10.1002/ajp.20394)

20. Digby L. 1995 Infant care, infanticide, and female reproductive strategies in polygynous groups of common marmosets (*Callithrix jacchus*). *Behav Ecol Sociobiol* 37, 51–61. (doi:10.1007/BF00173899)

21. Kirkpatrick-Tanne M, Aeschlimann C, Anzenberger G. 1996 Occurrence of an Infanticide within a Captive Polygynous Group of Common Marmosets, *Callithrix jacchus*. *Folia Primatologica* 67, 52–58. (doi:10.1159/000157206)

22. Lazaro-Perea C, Castro CSS, Harrison R, Araujo A, Arruda MF, Snowdon CT. 2000 Behavioral and demographic changes following the loss of the breeding female in cooperatively breeding marmosets. *Behav Ecol Sociobiol* 48, 137–146. (doi:10.1007/s002650000215)

23. Melo L, Mendes Pontes AR, Monteiro Da Cruz MAO. 2003 Infanticide and cannibalism in wild common marmosets. *Folia Primatologica* 74, 48–50. (doi:10.1159/000068389)

24. Roda SA, Pontes ARM. 1998 Polygyny and Infanticide in Common Marmosets in a Fragment of the Atlantic Forest of Brazil. *Folia Primatologica* 69, 372–376. (doi:10.1159/000021655)

25. Saltzman W, Digby LJ, Abbott DH. 2009 Reproductive skew in female common marmosets: What can proximate mechanisms tell us about ultimate causes? *Proceedings of the Royal Society B: Biological Sciences* 276, 389–399. (doi:10.1098/rspb.2008.1374)

26. Saltzman W, Abbott DH. 2005 Diminished maternal responsiveness during pregnancy in multiparous female common marmosets. *Horm Behav* 47, 151–163. (doi:10.1016/j.yhbeh.2004.10.001)

27. Saltzman W, Liedl KJ, Salper OJ, Pick RR, Abbott DH. 2008 Post-conception reproductive competition in cooperatively breeding common marmosets. *Horm Behav* 53, 274–286. (doi:10.1016/j.yhbeh.2007.10.005)

28. Yamamoto ME, Arruda M de F, Alencar AI, de Sousa MBC, Araújo A. 2009 Mating Systems and Female–Female Competition in the Common Marmoset, *Callithrix jacchus*. In *The Smallest Anthropoids*, pp. 119–133. Springer US. (doi:10.1007/978-1-4419-0293-1_6)

29. Tardif SD, Richter CB, Carson RL. 1984 Effects of sibling‐rearing experience on future reproductive success in two species of callitrichidae. *Am J Primatol* 6, 377–380. (doi:10.1002/ajp.1350060408)

30. Hernández AP, García GR. 2001 Retrospective Study of the Causes of Infant Death in a Common Marmoset (*Callithrix jacchus jacchus*) Colony. *Laboratory Primate Newsletter* 40, 1–4.

31. De Vleeschouwer K, Van Elsacker L, Leus K. 2001 Multiple breeding females in captive groups of golden-headed lion tamarins (*Leontopithecus chrysomelas*): causes and consequences. *Folia Primatol (Basel)* 72, 1–10. (doi:10.1159/000049913)

32. Herrera ERT, Knogge C, Heymann EW. 2000 Infanticide in a group of wild saddle-back tamarins, *Saguinus fuscicollis*. *Am J Primatol* 50, 153–157. (doi:10.1002/(sici)1098-2345(200002)50:2<153::aid-ajp5>3.0.co;2-%23)

33. Johnson LD, Ausman LM, Rolland RM, Chalifoux L V, Russell RG. 2001 Campylobacter-induced enteritis and diarrhea in captive cotton-top tamarins (*Saguinus oedipus*) during the first year of life. *Comp Med* 51, 257–261.

34. Leong KM, Terrell SP, Savage A. 2004 Causes of mortality in captive cotton-top tamarins (*Saguinus oedipus*). *Zoo Biol* 23, 127–137. (doi:10.1002/zoo.10121)

35. Savage A, Snowdon CT, Soto L, Medina F, Emeris G, Guillen R. 2021 Factors influencing the survival of wild cotton‐top tamarin (*Saguinus oedipus*) infants. *Am J Primatol* 83. (doi:10.1002/ajp.23262)

36. Evans S. 1983 Breeding of the cotton‐top tamarin *Saguinus oedipus oedipus*: A comparison with the common marmoset. *Zoo Biol* 2, 47–54. (doi:10.1002/zoo.1430020105)

37. Dronzek LA, Savage A, Snowdon CT, Whaling CS, Ziegler TE. 1986 Technique for hand-rearing and reintroducing rejected cotton-top tamarin infants. *Lab Anim Sci* 36, 243–247.

38. Johnson LD, Petto AJ, Sehgal PK. 1991 Factors in the rejection and survival of captive cotton top tamarins (*Saguinus oedipus*). *Am J Primatol* 25, 91–102. (doi:10.1002/ajp.1350250203)

39. Bardi M, Petto AJ, Lee-Parritz DE. 2001 Parental failure in captive cotton-top tamarins (*Saguinus Oedipus*). *Am J Primatol* 54, 159–169. (doi:10.1002/ajp.1020)

40. Kuhar CW, Bettinger TL, Sironen AL, Shaw JH, Lasley BL. 2003 Factors affecting reproduction in zoo-housed geoffroy’s tamarins (Saguinus geoffroyi). *Zoo Biol* 22, 545–559. (doi:10.1002/zoo.10099)

41. Culot L, Lledo-Ferrer Y, Hoelscher O, Muñoz Lazo FJJ, Huynen M-C, Heymann EW. 2011 Reproductive failure, possible maternal infanticide, and cannibalism in wild moustached tamarins, *Saguinus mystax*. *Primates* 52, 179–186. (doi:10.1007/s10329-011-0238-6)

42. Ramírez-Llorens P, Di Bitetti MS, Baldovino MC, Janson CH. 2008 Infanticide in black capuchin monkeys (*Cebus apella nigritus*) in Iguazú National Park, Argentina. *Am J Primatol* 70, 473–484. (doi:10.1002/ajp.20522)

43. Wirz A, Riviello MC. 2008 Reproductive parameters of a captive colony of capuchin monkeys (*Cebus apella*) from 1984 to 2006. *Primates* 49, 265–270. (doi:10.1007/s10329-008-0097-y)

44. Jack KM, Fedigan LM. 2009 Female dispersal in a female-philopatric species, *Cebus capucinus*. *Behaviour* 146, 471–497. (doi:10.1163/156853909X404420)

45. Fedigan LM, Jack KM. 2004 The demographic and reproductive context of male replacements in *Cebus capucinus*. *Behaviour* 141, 755–775. (doi:10.1163/1568539042245178)

46. Manson JH, Gros-Louis J, Perry S. 2004 Three apparent cases of infanticide by males in wild white-faced capuchins (*Cebus capucinus*). *Folia Primatologica* 75, 104–106. (doi:10.1159/000076270)

47. Schoof VAM, Wikberg EC, Jack KM, Fedigan LM, Ziegler TE, Kawamura S. 2014 Infanticides during Periods of Social Stability: Kinship, Resumption of Ovarian Cycling, and Mating Access in White-Faced Capuchins (*Cebus capucinus*). *Neotropical Primates* 21, 191–195. (doi:10.1896/044.021.0206)

48. Kulick NK, Cheves S, Chaves-Cordero C, Lopez R, Morales SR, Fedigan LM, Jack KM. 2021 Female-committed infanticide followed by juvenile-enacted cannibalism in wild white-faced capuchins. *Primates* 62, 1037–1043. (doi:s10329-021-00949-z)

49. Brasington LF, Wikberg EC, Kawamura S, Fedigan LM, Jack KM. 2017 Infant mortality in white-faced capuchins: The impact of alpha male replacements. *Am J Primatol* 79, 1–11. (doi:10.1002/ajp.22725)

50. Valderrama X, Srikosamatara S, Robinson JG. 1990 Infanticide in wedge-capped capuchin monkeys, *Cebus olivaceus*. *Folia Primatol (Basel)* 54, 171–176. (doi:10.1159/000156441)

51. Hopf S. 1981 Conditions of failure and recovery of maternal behavior in captive squirrel monkeys (Saimiri). *Int J Primatol* 2, 335–349. (doi:10.1007/BF02693483)

52. Hrdy SB. 1976 Care and Exploitation of Nonhuman Primate Infants by Conspecifics Other Than the Mother. *Adv Study Behav* 6, 101–158. (doi:10.1016/S0065-3454(08)60083-2)

53. Debyser IWJ. 1995 Platyrrhine juvenile mortality in captivity and in the wild. *Int J Primatol* 16, 909–933. (doi:10.1007/BF02696110)

54. Illia GA, Kowalewski M, Oklander LI. 2021 Evidence of an infanticide in black-horned capuchin monkeys (*Sapajus nigritus*) in an Atlantic Forest remnant in Argentina. *Notas sobre Mamíferos Sudamericanos* 03, 001–009. (doi:10.31687/saremNMS.21.2.6)

55. Busse CD, Gordon TP. 1983 Attacks on neonates by a male mangabey (*Cercocebus atys*). *Am J Primatol* 5, 345–356. (doi:10.1002/ajp.1350050404)

56. Maestripieri D, Wallen K, Carroll KA. 1997 Genealogical and demographic influences on infant abuse and neglect in group-living sooty mangabeys (*Cercocebus atys*). *Dev Psychobiol* 31, 175–180. (doi:10.1002/(SICI)1098-2302(199711)31:3<175::AID-DEV2>3.0.CO;2-P)

57. Fruteau C, Range F, Noë R. 2010 Infanticide risk and infant defence in multi-male free-ranging sooty mangabeys, *Cercocebus atys*. *Behavioural Processes* 83, 113–118. (doi:10.1016/j.beproc.2009.11.004)

58. Range F, Fo¨rderer T, Storrer-Meystre Y, Benetton C, Fruteau C. 2009 The structure of social relationships among sooty mangabeys in Taï. In *Monkeys of the Tai Forest* (eds WS McGraw, K Zuberbühler, R Noë), pp. 109–130. Cambridge: Cambridge University Press. (doi:10.1017/CBO9780511542121.005)

59. Kappeler PM, Fichtel C. 2012 A 15-year perspective on the social organization and life history of sifaka in Kirindy Forest. In *Long-Term Field Studies of Primates*, pp. 101–121. Berlin, Heidelberg: Springer Berlin Heidelberg. (doi:10.1007/978-3-642-22514-7_5)

60. Horrocks JA. 1986 Life-history characteristics of a wild population of vervets (*Cercopithecus aethiops sabaeus*) in Barbados, West Indies. *Int J Primatol* 7, 31–47. (doi:10.1007/BF02692308)

61. Isbell LA, Young TP, Jaffe KE, Carlson AA, Chancellor RL. 2009 Demography and life histories of sympatric patas monkeys, *Erythrocebus patas*, and vervets, *Cercopithecus aethiops*, in Laikipia, Kenyai. *Int J Primatol* 30, 103–124. (doi:10.1007/s10764-009-9332-7)

62. Kavanagh K, Dozier BL, Chavanne TJ, Fairbanks LA, Jorgensen MJ, Kaplan JR. 2011 Fetal and maternal factors associated with infant mortality in vervet monkeys. *J Med Primatol* 40, 27–36. (doi:10.1111/j.1600-0684.2010.00441.x)

63. Angst W, Thommen D. 1977 New Data and a Discussion of Infant Killing in Old World Monkeys and Apes. *Folia Primatologica* 27, 198–229. (doi:10.1159/000155787)

64. Struhsaker TT. 1977 Infanticide and social organization in the redtail monkey (*Ceraopithecus ascanius schmidti*) in the Kibale forest, Uganda. *New York Zoological Society and Rockefeller University* 45, 75–84.

65. Kane EE, Gnépa F. 2016 An Infanticide Attempt After Male Takeover in Diana Monkeys (*Cercopithecus diana diana*) in Taï, Côte d’Ivoire. *Afr Primates* 11, 37–40.

66. Butynski TM. 1990 Comparative ecology of blue monkeys (*Cercopithecus mitis*) in high- and low-density subpopulations. *Ecol Monogr* 60, 1–26. (doi:10.2307/1943024)

67. Böer M, Sommer V. 1992 Evidence for sexually selected infanticide in captive *Cercopithecus mitis*, *Cercocebus torquatus*, and *Mandrillus leucophaeus*. *Primates* 33, 557–563. (doi:10.1007/BF02381156)

68. Cords M, Fuller JL. 2010 Infanticide in *Cercopithecus mitis stuhlmanni* in the Kakamega Forest, Kenya: Variation in the Occurrence of an Adaptive Behavior. *Int J Primatol* 31, 409–431. (doi:10.1007/s10764-010-9400-z)

69. Fairgrieve C. 1995 Infanticide and Infant Eating in the Blue Monkey (*Cercopithecus mitis stuhlmanni*) in the Budongo Forest Reserve, Uganda. *Folia Primatologica* 64, 69–72. (doi:10.1159/000156835)

70. Isbell LA, Cheney DL, Seyfarth RD. 2002 Why vervet monkeys (*Cercopithecus aethiops*) live in multimale groups. *The Guenons: Diversity and Adaptation in African Monkeys* , 173–187. (doi:10.1007/0-306-48417-x_13)

71. Fairbanks LA, Mcguir MT, Mcguir MT. 1995 Maternal Condition and the Quality of Maternal Care in Vervet Monkeys. *Behaviour* 132, 733–754. (doi:10.1163/156853995X00126)

72. Fairbanks LA. 1996 Individual Differences in Maternal Style. Causes and Consequences for Mothers and offspring. *Adv Study Behav* 25, 579–611. (doi:10.1016/S0065-3454(08)60343-5)

73. Harris TR, Monfort SL. 2003 Behavioral and Endocrine Dynamics Associated with Infanticide in a Black and White Colobus Monkey (*Colobus guereza*). *Am J Primatol* 61, 135–142. (doi:10.1002/ajp.10116)

74. Ondherdonk DA. 2000 Infanticide of a newborn black-and-white colobus monkey (*Colobus guereza*) in Kibale National Park, Uganda. *Primates* 41, 209–212. (doi:10.1007/bf02557802)

75. Sicotte P, Teichroeb JA, Saj TL. 2007 Aspects of male competition in *Colobus vellerosus*: Preliminary data on male and female loud calling, and infant deaths after a takeover. *Int J Primatol* 28, 627–636. (doi:10.1007/s10764-007-9141-9)

76. Sicotte P, Teichroeb J. 2008 Infanticide in ursine colobus monkeys (*Colobus vellerosus*) in Ghana: new cases and a test of the existing hypotheses. *Behaviour* 145, 727–755. (doi:10.1163/156853908783929160)

77. Goodwin RM, Ota RC. 2020 Aggressive Intergroup Encounter and Infanticide in the White-thighed Colobus (*Colobus vellerosus*) at Kikélé Sacred Forest, Bénin. *Afr Primates* 14, 45–50.

78. Enstam KL, Isbell LA, Maar TW De. 2002 Male Demography, Female Mating Behavior, and Infanticide in Wild Patas Monkeys (*Erythrocebus patas*). *Int J Primatol* 103, 239–248. (doi:0164-0291/02/0200-0085)

79. Brown M. 2020 An observation of intergroup infanticide in grey-cheeked mangabeys (*Lophocebus albigena*). *Behaviour* 157, 1091–1098. (doi:10.1163/1568539X-bja10031)

80. Solanki GS, Zothansiama. 2013 Infanticide in captive stump-tailed macaques (*Macaca arctoides*) is in accordance with the sexual selection hypothesis. *Curr Sci* 104, 1081–1083.

81. De Ruiter JR, Van Hooff JARAM, Scheffrahn W. 1994 Social and Genetic Aspects of Paternity in Wild Long-Tailed Macaques (*Macaca Fascicularis*). *Behaviour* 129, 203–224. (doi:10.1163/156853994X00613)

82. Honjo S, Chop F, Fujiwara T, Yoshioka Y, Masuko K, Kurihara K, Yabe M, Noguchi Y. 1978 Breeding of cynomolgus monkeys through successive generations by indoor cage system. *Jpn J Med Sci Biol* 31, 301–310. (doi:10.7883/yoken1952.31.301)

83. Luder HU. 1993 Hazard rates and causes of death in a captive group of crab‐eating monkeys (*Macaca fascicularis*). *Am J Primatol* 30, 139–147. (doi:10.1002/ajp.1350300206)

84. Fedigan LM, Zohar S. 1997 Sex differences in mortality of Japanese macaques: Twenty-one years of data from the Arashiyama West population. *Am J Phys Anthropol* 102, 161–175. (doi:10.1002/(SICI)1096-8644(199702)102:2<161::AID-AJPA2>3.0.CO;2-1)

85. Breslin WJ, Hilbish KG, Martin JA, Halstead CA, Newcomb DL, Chellman GJ. 2015 An Enhanced Pre- and Postnatal Development Study in Cynomolgus Monkeys with Tabalumab: A Human IgG4 Monoclonal Antibody. *Birth Defects Research (Part B)* 116, 100–116. (doi:10.1002/bdrb.21146)

86. Levallois L, De Marigny SD. 2015 Reproductive success of wild-caught and captive-bred cynomolgus macaques at a breeding facility. *Lab Anim (NY)* 44, 387–393. (doi:10.1038/laban.733)

87. Widayati K, Noerwana O, Fauzi R, Tsuji Y. 2022 Case Report of Infanticide by a Wild Long-tailed Macaque (*Macaca Fascicularis*). *Philipp J Sci* 151, 751–753. (doi:10.56899/151.02.17)

88. Quiatt D. 1979 Aunts and Mothers: Adaptive Implications of Allomaternal Behavior of Nonhuman Primates. *Am Anthropol* 81, 310–319. (doi:10.1525/aa.1979.81.2.02a00040)

89. Troisi A, D’Amato FR. 1984 Ambivalence in monkey mothering: Infant abuse combined with maternal possessiveness. *Journal of Nervous and Mental Disease* 172, 105–108. (doi:10.1097/00005053-198402000-00007)

90. Yamada K, Nakamichi M. 2006 A fatal attack on an unweaned infant by a non-resident male in a free-ranging group of Japanese macaques (*Macaca fuscata*) at Katsuyama. *Primates* 47, 165–169. (doi:10.1007/s10329-005-0154-8)

91. Soltis J, Thomsen R, Matsubayashi K, Takenaka O. 2000 Infanticide by resident males and female counter-strategies in wild Japanese macaques (*Macaca fuscata*). *Behav Ecol Sociobiol* 48, 195–202. (doi:10.1007/s002650000224)

92. Hiraiwa M. 1981 Maternal and alloparental care in a troop of free-ranging Japanese monkeys. *Primates* 22, 309–329. (doi:10.1007/BF02381573)

93. Majolo B, Schino G, Troisi A. 2005 Towards thirty years of ethological research on the Japanese macaque (*Macaca fuscata*) colony of the Rome Zoo: a review. *Journal of Anthropological Sciences* 83, 43–60.

94. Pflüger LS, Pink KE, Wallner B, Radler C, Dorner M, Huffman MA. 2021 Twenty-three-year demographic history of the Affenberg Japanese macaques (*Macaca fuscata*), a translocated semi-free-ranging group in southern Austria. *Primates* 62, 761–776. (doi:10.1007/s10329-021-00928-4)

95. Ruppenthal GC. 1976 A 10-year perspective of motherless-mother monkey behavior. *J Abnorm Psychol* 85, 341–349. (doi:10.1037/0021-843X.85.4.341)

96. Bahr NI, Pryce CR, Döbeli M, Martin RD. 1998 Evidence from urinary cortisol that maternal behavior is related to stress in gorillas. *Physiol Behav* 64, 429–437. (doi:10.1016/S0031-9384(98)00057-2)

97. Dyke B, Gage TB, Mamelka PM, Goy RW, Stone WH. 1986 A demographic analysis of the Wisconsin Regional Primate Center rhesus colony, 1962–1982. *Am J Primatol* 10, 257–269. (doi:10.1002/ajp.1350100306)

98. Goin DL, Gust DA. 1998 Peer-rearing influences subsequent maternal behavior and infant survival in a newly formed herpes B-virus negative rhesus macaque group. *Primates* 39, 539–543. (doi:10.1007/BF02557574)

99. Maestripieri D. 2005 Early experience affects the intergenerational transmission of infant abuse in rhesus monkeys. *Proc Natl Acad Sci U S A* 102, 9726–9729. (doi:10.1073/pnas.0504122102)

100. Maestripieri D, Higley JD, Lindell SG, Newman TK, McCormack KM, Sanchez MM. 2006 Early maternal rejection affects the development of monoaminergic systems and adult abusive parenting in rhesus macaques (*Macaca mulatta*). *Behavioral Neuroscience* 120, 1017–1024. (doi:10.1037/0735-7044.120.5.1017)

101. Maestripieri D, Carroll KA. 1998 Risk factors for infant abuse and neglect in group-living rhesus monkeys. *Psychol Sci* 9, 143–145. (doi:10.1111/1467-9280.00027)

102. Maestripieri D, Tomaszycki M, Carroll KA. 1999 Consistency and change in the behavior of rhesus macaque abusive mothers with successive infants. *Dev Psychobiol* 34, 29–35. (doi:10.1002/(SICI)1098-2302(199901)34:1<29::AID-DEV5>3.0.CO;2-U)

103. Numan M, Insel TR. 2003 Experiential Factors Influencing Maternal Behavior. In *The Neurobiology of Parental Behavior*, pp. 42–68. Springer New York. (doi:10.1007/0-387-21799-1_3)

104. Field T, Reite M. 1985 The Psychobiology of Attachment and Separation: A Summary. In *The Psychobiology of Attachment and Separation*, pp. 455–479. (doi:10.1016/b978-0-12-586780-1.50020-3)

105. Troisi A, D’Amato FR. 1983 Is monkey maternal abuse of offspring aggressive behavior? *Aggress Behav* 9, 167–173. (doi:10.1002/1098-2337(1983)9:2<167::AID-AB2480090207>3.0.CO;2-X)

106. Ciani AC. 1984 A case of infanticide in a free-ranging group of rhesus monkeys (*Macaca mulatta*) in the Jackoo. *Primates* 25, 372–377.

107. Clarke MR, Blanchard JL, Snyder JA. 1995 Infant-killing in Pigtailed Monkeys: A Colony Management Concern. *Laboratory Primate Newsletter* 34, 2–5. (doi:10.1002/9781119179313.wbprim0298)

108. Maestripieri D, Carroll KA. 1998 Behavioral and environmental correlates of infant abuse in group-living pigtail macaques. *Infant Behav Dev* 21, 603–612. (doi:10.1016/S0163-6383(98)90032-7)

109. Maestripieri D, Wallen K. 1995 Interest in infants varies with reproductive condition in group-living female pigtail macaques (*Macaca nemestrina*). *Physiol Behav* 57, 353–358. (doi:10.1016/0031-9384(94)00222-Q)

110. Maestripieri D, Wallen K, Carroll KA. 1997 Infant abuse runs in families of group-living pigtail macaques. *Child Abuse Negl* 21, 465–471. (doi:10.1016/S0145-2134(97)00006-9)

111. Singh M, Kumara HN, Kumar MA, Singh M, Cooper M. 2006 Male influx, infanticide, and female transfer in *Macaca radiata radiata*. *Int J Primatol* 27, 515–528. (doi:10.1007/s10764-006-9031-6)

112. Redman HC, Schneider R. 1979 An epidemiological study of neonatal and postneonatal mortality in *Macaca radiata* at the California Primate Research Center (1966-1973). *J Med Primatol* 8, 1–17. (doi:10.1159/000460171)

113. Hsu MJ, Lin J-F, Agoramoorthy G. 2000 Occurrence of Twins in Wild Formosan Macaques, *Macaca cyclopis*, at Mt. Longevity, Taiwan. *Folia Primatologica* 71, 154–156. (doi:10.1159/000021745)

114. Paul A, Kuester J. 1996 Infant handling by female barbary macaques (*Macaca sylvanus*) at Affenberg Salem: Testing functional and evolutionary hypotheses. *Behav Ecol Sociobiol* 39, 133–145. (doi:10.1007/s002650050275)

115. Berman CM, Li J, Ogawa H, Ionica C, Yin H. 2007 Primate Tourism, Range Restriction, and Infant Risk Among *Macaca thibetana* at Mt. Huangshan, China. *Int J Primatol* 28, 1123–1141. (doi:10.1007/s10764-007-9199-4)

116. Muroyama Y, Thierry B. 1996 Fatal attack on an infant by an adult female Tonkean macaque. *Int J Primatol* 17, 219–227. (doi:10.1007/BF02735449)

117. Agoramoorthy G, Hsu MJ. 2005 Occurrence of infanticide among wild proboscis monkeys (*Nasalis larvatus*) in Sabah, Northern Borneo. *Folia Primatologica* 76, 177–179. (doi:10.1159/000084380)

118. Henzi P, Barrett L. 2003 Evolutionary Ecology, Sexual Conflict, and Behavioral Differentiation Among Baboon Populations. *Evol Anthropol* 12, 217–230. (doi:10.1002/evan.10121)

119. Palombit R a. 2003 Male infanticide in wild savanna baboons: Adaptive significance and intraspecific variation. *Sexual selection and reproductive competition in primates: new perspectives and directions* , 3–47.

120. Neal SJ, Schapiro SJ, Lambeth SP, Magden ER. 2024 Nursery- vs. Mother-Reared Baboons: Reproductive Success and Health Parameters. *Vet Sci* 11, 416. (doi:10.3390/vetsci11090416)

121. Shopland JM. 1982 An intergroup encounter with fatal consequences in yellow baboons (*Papio cynocephalus*). *Am J Primatol* 3, 263–266. (doi:10.1002/ajp.1350030123)

122. Kleindorfer S, Wasser SK. 2004 Infant handling and mortality in yellow baboons (*Papio cynocephalus*): Evidence for female reproductive competition? *Behav Ecol Sociobiol* 56, 328–337. (doi:10.1007/s00265-004-0798-1)

123. Shopland JM, Altmann J. 1987 Fatal intragroup kidnapping in yellow baboons. *Am J Primatol* 13, 61–65. (doi:10.1002/ajp.1350130108)

124. Gomendio M, Colmenares F. 1989 Infant Killing and Infant Adoption Following the Introduction of New Males to an All‐Female Colony of Baboons. *Ethology* 80, 223–244. (doi:10.1111/j.1439-0310.1989.tb00742.x)

125. Rijksen HD. 1981 Infant killing: a possible consequence of a disputed leader role. *Behaviour* 78, 138–167. (doi:10.1163/156853981x00293)

126. Swedell L, Tesfaye T. 2003 Infant mortality after takeovers in wild Ethiopian hamadryas baboons. *Am J Primatol* 60, 113–118. (doi:10.1002/ajp.10096)

127. Swedell L. 2000 Two takeovers in wild hamadryas baboons. *Folia Primatologica* 71, 169–172. (doi:10.1159/000021736)

128. Zinner D. 1993 Infant mortality in captive Hamadryas baboons (*Papio hamadryas*). *Primate Rep* 36, 97.

129. Zinner D, Deschner T. 2000 Sexual swellings in female hamadryas baboons after male take-overs: ‘Deceptive’ swellings as a possible female counter-strategy against infanticide. *Am J Primatol* 52, 157–168. (doi:10.1002/1098-2345(200012)52:4<157::AID-AJP1>3.0.CO;2-L)

130. Amann AL, Pines M, Swedell L. 2017 Contexts and consequences of takeovers in hamadryas baboons: Female parity, reproductive state, and observational evidence of pregnancy loss. *Am J Primatol* 79, 1–8. (doi:10.1002/ajp.22649)

131. Brent L, Koban T, Ramirez S. 2002 Abnormal, abusive, and stress-related behaviors in baboon mothers. *Biol Psychiatry* 52, 1047–1056. (doi:10.1016/S0006-3223(02)01540-8)

132. Sunderland N, Heffernan S, Thomson S, Hennessy A. 2008 Maternal parity affects neonatal survival rate in a colony of captive bred baboons (*Papio hamadryas*). *J Med Primatol* 37, 223–228. (doi:10.1111/j.1600-0684.2007.00277.x)

133. Waters SS. 1995 A review of social parameters which influence breeding Pithecia pithecia in White‐faced saki in captivity. *International Zoo Yearbook* 34, 147–153. (doi:10.1111/j.1748-1090.1995.tb00673.x)

134. Winans JC, Learn NH, Long’ida Siodi I, Kinyua Warutere J, Archie EA, Tung J, Alberts SC, Markham AC. 2024 High early lactational synchrony within baboon groups predicts increased female-female competition and infant mortality. (doi:10.1101/2024.09.09.611196)

135. Birrell AM, Hennessy A, Gillin A, Horvath J, Tiller D. 1996 Reproductive and neonatal outcomes in captive bred baboons (*Papio hamadryas*). *J Med Primatol* 25, 287–293. (doi:10.1111/j.1600-0684.1996.tb00212.x)

136. Rijksen HD. 1981 Infant Killing; A Possible Consequence of a Disputed. *Behaviour* 78, 138–168. (doi:https://www.jstor.org/stable/4534134)

137. Brain C. 1992 Deaths in a desert baboon troop. *Int J Primatol* 13, 593–599. (doi:10.1007/BF02551255)

138. Collins DA, Curt D. Busse, Jane Goodall. 1984 Infanticide in two populations of savanna baboons. In *Infanticide: Comparative and Evolutionary Perspectives* (eds Glenn Hausfater, Sarah Blaffer Hrdy), pp. 193–216. Routledge. (doi:10.4324/9780203788608)

139. Zipple MN, Grady JH, Gordon JB, Chow LD, Archie EA, Altmann J, Alberts SC. 2017 Conditional fetal and infant killing by male baboons. *Proceedings of the Royal Society B: Biological Sciences* 284. (doi:10.1098/rspb.2016.2561)

140. Huchard E, Alvergne A, Féjan D, Knapp LA, Cowlishaw G, Raymond M. 2010 More than friends? Behavioural and genetic aspects of heterosexual associations in wild chacma baboons. *Behav Ecol Sociobiol* 64, 769–781. (doi:10.1007/s00265-009-0894-3)

141. Palombit RA, Seyfarth RM, Cheney DL. 1997 The adaptive value of ‘friendships’ to female baboons: experimental and observational evidence. *Anim Behav* 54, 599–614. (doi:10.1006/anbe.1996.0457)

142. Weingrill T. 2000 Infanticide and the value of male-female relationships in mountain chacma baboons. *Behaviour* 5, 150–150. (doi:10.1136/jme.5.3.150)

143. Starin ED. 1994 Philopatry and affiliation among Red Colobus. *Behaviour* 130, 253–270. (doi:10.1163/156853994X00550)

144. Marsh CW. 1979 Comparative Aspects of Social Organization in the Tana River Red Colobus, *Colobus badius rufomitratus*. *Z Tierpsychol* 51, 337–362. (doi:10.1111/j.1439-0310.1979.tb00695.x)

145. Struhsaker TT, Leland L. 1985 Infanticide in a Patrilineal Society of Red Colobus Monkeys. *Z Tierpsychol* 69, 89–132. (doi:10.1111/j.1439-0310.1985.tb00139.x)

146. Xiang Z, Grueter CC. 2007 First direct evidence of infanticide and cannibalism in wild snub-nosed monkeys (*Rhinopithecus bieti*). *Am J Primatol* 69, 249–254. (doi:10.1002/ajp.20333)

147. Zhang S, Liang B, Wang L. 1999 Infanticide within Captive Groups of Sichuan Golden Snub-Nosed Monkeys (*Rhinopithecus roxellana*). *Folia Primatologica* 70, 274–276. (doi:10.1159/000021707)

148. Li W *et al.* 2024 Infanticide in golden snub-nosed monkeys with multilevel society. *Curr Zool*. 70, 273–275. (doi:10.1093/cz/zoad007)

149. Yao H, Yu H, Yang B, Yang W, Xu H, Grueter CC, Li M, Xiang Z. 2016 Male Infanticide in the Golden Snub-Nosed Monkey (*Rhinopithecus roxellana*), a Seasonally Breeding Primate. *Int J Primatol* 37, 175–184. (doi:10.1007/s10764-016-9892-2)

150. Borries C. 1997 Infanticide in seasonally breeding multimale groups of Hanuman langurs (*Presbytis entellus*) in Ramnagar (South Nepal). *Behav Ecol Sociobiol* 41, 139–150. (doi:10.1007/s002650050373)

151. Hrdy SB. 1974 Male-Male Competition and Infanticide Among the Langurs (*Presbytis Entellus*) Rajasthan. *Folia Primatologica* 22, 19–58. (doi:10.1159/000155616)

152. Newton PN. 1986 Infanticide in an undisturbed forest population of hanuman langurs, *Presbytis enteilus*. *Anim Behav* 8, 785-789 Infanticide. (doi:10.1007/BF02735173)

153. Ross C. 1993 Take‐over and infanticide in South Indian Hanuman langurs (*Presbytis entellus*). *Am J Primatol* 30, 75–82. (doi:10.1002/ajp.1350300106)

154. Singh A, Sharma G, Rajpurohit LS. 2018 Male Bias Infanticide after Resident Male Replacement in *Semnopithecus entellus* Around Jodhpur (India). *International Journal of Research and Analytical Reviews*

155. Mohnot SM. 1980 Intergroup Infant Kidnapping in Hanuman Langur. *Folia Primatologica* 34, 259–277. (doi:10.1159/000155958)

156. Kavana TS, Erinjery JJ, Singh M. 2014 Male Takeover and Infanticide in Nilgiri langurs *Semnopithecus johnii* in the Western Ghats, India. *Folia Primatologica* 85, 164–177. (doi:10.1159/000362546)

157. Salmi R, Lu A, Hofner AN, Madushan C, Thisaru D, Mallott EK, Vandercone R. 2024 Male Infanticide in the Northern Purple-Faced Langur (*Semnopithecus vetulus Philbricki*) in the Kaludiyapukuna Forest Reserve, Sri Lanka. *Am J Primatol* 86. (doi:10.1002/ajp.23693)

158. Beehner JC, Bergman TJ. 2008 Infant mortality following male takeovers in wild geladas. *Am J Primatol* 70, 1152–1159. (doi:10.1002/ajp.20614)

159. Moos R, Rock J, Salzert W. 1985 Infanticide in gelada baboons (*Theropithecus gelada*). *Primates* 26, 497–500. (doi:10.1007/BF02382465)

160. Mori A, Belay G, Iwamoto T. 2003 Changes in unit structures and infanticide observed in Arsi geladas. *Primates* 44, 217–223. (doi:10.1007/s10329-002-0013-9)

161. Mori A, Iwamoto T, Bekele A. 1997 A case of infanticide in a recently found gelada population in Arsi, Ethiopia. *Primates* 38, 79–88. (doi:10.1007/BF02385924)

162. Kifle Z. 2024 Infanticide by male gelada leader and group counterstrategies. *Afr J Ecol* 62. (doi:10.1111/aje.13169)

163. Shelmidine N, Mcaloose D, Mccann C. 2013 Survival patterns and mortality in the North American population of silvered leaf monkeys (*Trachypithecus cristatus*). *Zoo Biol* 32, 177–188. (doi:10.1002/zoo.21055)

164. Qi-Hai Z, Bang L, Cheng-Ming H. 2013 Attacks on adult females with infants by non-resident males in the François langur. *Zool Res* 34, E9. (doi:10.3724/SP.J.1141.2013.E01E09)

165. YIN L, JIN T, WATANABE K, QIN D, WANG D, PAN W. 2013 Male attacks on infants and infant death during male takeovers in wild white‐headed langurs (*Trachypithecus leucocephalus*). *Integr Zool* 8, 365–377. (doi:10.1111/1749-4877.12022)

166. Stangerl KF’, Coffman’ BS, Kay M. 1995 Reproduction in Coquerel’s Dwarf Lemur (*Mirza coquereli*). *Am J Primatol*.

167. Haring DM, Hess WR, Coffman BS, Simons EL, Owens TM. 1994 Natural history and captive management of the aye-aye *Daubentonia madagascariensis* at the Duke University Primate Center, Durham. *International zoo yearbook. Vol. 33* 1, 201–219. (doi:10.1111/j.1748-1090.1993.tb00624.x)

168. Debyser IWJ. 1995 Prosimian juvenile mortality in zoos and primate centers. *Int J Primatol* 16, 889–907. (doi:10.1007/BF02696109)

169. Izard MK, Simons EL. 1986 Isolation of females prior to parturition reduces neonatal mortality in Galago. *Am J Primatol* 10, 249–255. (doi:10.1002/ajp.1350100305)

170. Yamagiwa J, Kahekwa J, Basabose AK. 2009 Infanticide and social flexibility in the genus Gorilla. *Primates* 50, 293–303. (doi:10.1007/s10329-009-0163-0)

171. Robbins AM, Gray M, Basabose A, Uwingeli P, Mburanumwe I, Kagoda E, Robbins MM. 2013 Impact of male infanticide on the social structure of mountain gorillas. *PLoS One* 8. (doi:10.1371/journal.pone.0078256)

172. Robbins MM. 1995 A Demographic Analysis of Male Life History and Social Structure of Mountain Gorillas. *Behaviour* 132, 21–47. (doi:10.1163/156853995X00261)

173. Stokes EJ, Parnell RJ, Olejniczak C. 2003 Female dispersal and reproductive success in wild western lowland gorillas (*Gorilla gorilla gorilla*). *Behav Ecol Sociobiol* 54, 329–339. (doi:10.1007/s00265-003-0630-3)

174. Watts DP. 1989 Infanticide in Mountain Gorillas: New Cases and a Reconsideration of the Evidence. *Ethology* 81, 1–18. (doi:10.1111/j.1439-0310.1989.tb00754.x)

175. Robbins AM, Gray M, Uwingeli P, Mburanumwe I, Kagoda E, Robbins MM. 2014 Variance in the reproductive success of dominant male mountain gorillas. *Primates* 55, 489–499. (doi:10.1007/s10329-014-0426-2)

176. Cole RE. 2000 Obstetric management of a protracted labor in a captive Western lowland gorilla. In *American Journal of Obstetrics and Gynecology*, pp. 1306–1311. (doi:10.1067/mob.2000.106185)

177. Kralick AE *et al.* 2017 A radiographic study of permanent molar development in wild Virunga mountain gorillas of known chronological age from R wanda. *Am J Phys Anthropol* 163, 129–147. (doi:10.1002/ajpa.23192)

178. Arcadi AC, Wrangham RW. 1999 Infanticide in Chimpanzees: Review of cases and a new within-group observation from the Kanyawara study group in Kibale National Park. *Primates* 40, 337–351. (doi:10.1007/BF02557557)

179. Bakuneeta C, Inagaki H, Reynolds V. 1993 Identification of wild chimpanzee hair samples from feces by electron microscopy. *Primates* 34, 233–235. (doi:10.1007/BF02381396)

180. Boesch C, Crockford C, Herbinger I, Wittig R, Moebius Y, Normand E. 2008 Intergroup conflicts among chimpanzees in Taï National Park: Lethal violence and the female perspective. *Am J Primatol* 70, 519–532. (doi:10.1002/ajp.20524)

181. Bygott JD. 1972 Cannibalism among wild Chimpanzees. *Nature* 238, 410–411. (doi:10.1038/238410a0)

182. Goodall J. 1977 Infant Killing and Cannibalism in Free-Living Chimpanzees. *Folia Primatologica* 28, 259–282. (doi:10.1159/000155817)

183. Hamai M, Nishida T, Takasaki H, Turner LA. 1992 New records of within-group infanticide and cannibalism in wild chimpanzees. *Primates* 33, 151–162. (doi:10.1007/BF02382746)

184. Kawanaka K. 1981 Infanticide and cannibalism in chimpanzees: with special reference to the newly observed case in the Mahale mountains. *Afr Study Monogr* 1, 69–99.

185. Kutsukake N, Matsusaka T. 2002 Incident of intense aggression by chimpanzees against an infant from another group in Mahale Mountains National Park, Tanzania. *Am J Primatol* 58, 175–180. (doi:10.1002/ajp.10058)

186. Murray CM, Wroblewski E, Pusey AE. 2007 New case of intragroup infanticide in the chimpanzees of Gombe National Park. *Int J Primatol* 28, 23–37. (doi:10.1007/s10764-006-9111-7)

187. Newton-Fisher NE. 1999 Infant killers of Budongo. *Folia Primatologica* 70, 167–169. (doi:10.1159/000021690)

188. Nishida T, Uehara S, Nyundo R. 1979 Predatory behavior among wild chimpanzees of the mahale mountains. *Primates* 20, 1–20. (doi:10.1007/BF02373826)

189. Nishida T, Kawanaka K. 1985 Within-group cannibalism by adult male chimpanzees. *Primates* 26, 274–284. (doi:10.1007/BF02382402)

190. Nishida T *et al.* 2003 Demography, female life history, and reproductive profiles among the chimpanzees of Mahale. *Am J Primatol* 59, 99–121. (doi:10.1002/ajp.10068)

191. Norikoshi K. 1982 One observed case of cannibalism among wild chimpanzees of the Mahale mountains. *Primates* 23, 66–74. (doi:10.1007/BF02381438)

192. Sherrow HM, Amsler SJ. 2007 New intercommunity infanticides by the chimpanzees of Ngogo, Kibale National Park, Uganda. *Int J Primatol* 28, 9–22. (doi:10.1007/s10764-006-9112-6)

193. Spijkerman RP, Van Hooff JARAM, Jens W. 1990 A case of lethal infant abuse in an established group of chimpanzees. *Folia Primatologica* 55, 41–44. (doi:10.1159/000156496)

194. Suzuki A. 1971 Carnivority and cannibalism observed among forest-living chimpanzees. *Journal of the Anthropological Society of Nippon*, 30–48.

195. Takahata Y. 1985 Adult male chimpanzees kill and eat a male newborn infant: Newly observed intragroup infanticide and cannibalism in mahale national park, Tanzania. *Folia Primatologica* 44, 161–170. (doi:10.1159/000156210)

196. Watts DP, Mitani JC. 2000 Infanticide and cannibalism by male chimpanzees at Ngogo, Kibale National Park, Uganda. *Primates* 41, 357–365. (doi:10.1007/BF02557646)

197. Watts DP, Sherrow HM, Mitani JC. 2002 New cases of inter-community infanticide by male Chimpanzees at Ngogo, Kibale National Park, Uganda. *Primates* 43, 263–270. (doi:10.1007/BF02629601)

198. Williams JM, Lonsdorf E V., Wilson ML, Schumacher‐Stankey J, Goodall J, Pusey AE. 2008 Causes of death in the Kasekela chimpanzees of Gombe National Park, Tanzania. *Am J Primatol* 70, 766–777. (doi:10.1002/ajp.20573)

199. Wilson ML, Wallauer WR, Pusey AE. 2004 New Cases of Intergroup Violence Among Chimpanzees in Gombe National Park, Tanzania. *Int J Primatol* 25, 50–62. (doi:10.1023/B)

200. Kirchhoff CA, Wilson ML, Mjungu DC, Raphael J, Kamenya S, Collins DA. 2018 Infanticide in chimpanzees: Taphonomic case studies from Gombe. *Am J Phys Anthropol* 165, 108–122. (doi:10.1002/ajpa.23335)

201. Leroux M *et al.* 2022 First observation of a chimpanzee with albinism in the wild: Social interactions and subsequent infanticide. *Am J Primatol* 84, 1–7. (doi:10.1002/ajp.23305)

202. Nishie H, Nakamura M. 2018 A newborn infant chimpanzee snatched and cannibalized immediately after birth: Implications for “maternity leave” in wild chimpanzee. *Am J Phys Anthropol* 165, 194–199. (doi:10.1002/ajpa.23327)

203. Lowe AE, Hobaiter C, Asiimwe C, Zuberbühler K, Newton-Fisher NE. 2019 Intra-community infanticide in wild, eastern chimpanzees: a 24-year review. *Primates* 61, 69–82. (doi:10.1007/s10329-019-00730-3)

204. Goodall J. 1986 *The Chimpanzees of Gombe: Patterns of Behavior.* Cambridge: Belknap Press.

205. Goodall J. 1983 Population Dynamics during a 15 Year Period in one Community of Free‐living Chimpanzees in the Gombe National Park, Tanzania. *Z Tierpsychol* 61, 1–60. (doi:10.1111/j.1439-0310.1983.tb01324.x)

206. Pusey A, Murray C, Wallauer W, Wilson M, Wroblewski E, Goodall J. 2008 Severe aggression among female *Pan troglodytes schweinfurthii* at Gombe National Park, Tanzania. *Int J Primatol* 29, 949–973. (doi:10.1007/s10764-008-9281-6)

207. Townsend SW, Slocombe KE, Emery Thompson M, Zuberbühler K. 2007 Female-led infanticide in wild chimpanzees. *Current Biology* 17, R355–R356. (doi:10.1016/j.cub.2007.03.020)

208. Walker CS, Walker KK, Paulo G, Pusey AE. 2018 Morphological identification of hair recovered from feces for detection of cannibalism in eastern chimpanzees. *Folia Primatologica* 89, 240–250. (doi:10.1159/000488509)

209. Walker KK, Foerster S, Murray CM, Mjungu D, Pusey AE. 2021 Evaluating adaptive hypotheses for female-led infanticide in wild chimpanzees. *Anim Behav* 180, 23–36. (doi:10.1016/j.anbehav.2021.07.025)

210. Grilly DM, Ferraro DP, Braude MC. 1974 Observations on the Reproductive Activity of Chimpanzees Following Long-term Exposure to Marihuana. *Pharmacology* 11, 304–307. (doi:10.1159/000136502)

211. Hobson WC, Graham CE, Rowell TJ. 1991 National chimpanzee breeding program: Primate research institute. *Am J Primatol* 24, 257–263. (doi:10.1002/ajp.1350240311)

212. Siebert JR, Swindler DR. 1991 Perinatal dental development in the chimpanzee (*Pan troglodytes*). *Am J Phys Anthropol* 86, 287–294. (doi:10.1002/ajpa.1330860215)

213. Markham RJ. 1990 Breeding orangutans at Perth Zoo: Twenty years of appropriate husbandry. *Zoo Biol* 9, 171–182. (doi:10.1002/zoo.1430090212)

214. Knott CD, Scott AM, O’Connell CA, Scott KS, Laman TG, Riyandi, Susanto TW. 2019 Possible Male Infanticide in Wild Orangutans and a Re-evaluation of Infanticide Risk. *Sci Rep* 9, 1–16. (doi:10.1038/s41598-019-42856-w)

215. Alfred JRB, Sati JP. 1991 On the first record of infanticide in the Hoolock gibbon *Hylobates hoolock* in the wild. *Rec Zool Surv India* 89, 319–321–319–321.

216. Borries C, Savini T, Koenig A. 2011 Social monogamy and the threat of infanticide in larger mammals. *Behav Ecol Sociobiol* 65, 685–693. (doi:10.1007/s00265-010-1070-5)

217. Mootnick AR, Baker E, Sheeran LK. 2005 Familiarity during immaturity: Implications for the captive propagation of gibbons. *Int J Primatol* 26, 1417–1433. (doi:10.1007/s10764-005-8860-z)

218. Lewis RJ, Razafindrasamba SM, Tolojanahary JP. 2003 Observed infanticide in a seasonal breeding prosimian (*Propithecus verreauxi verreauxi*) in Kirindy Forest, Madagascar. *Folia Primatologica* 74, 101–103. (doi:10.1159/000070006)

219. Littlefield BL. 2010 Infanticide following male takeover event in Verreaux’s sifaka (*Propithecus verreauxi verreauxi*). *Primates* 51, 83–86. (doi:10.1007/s10329-009-0162-1)

220. Morelli TL, King SJ, Pochron ST, Wright PC. 2009 The rules of disengagement: Takeovers, infanticide, and dispersal in a rainforest lemur, Propithecus edwardsi. *Behaviour* 146, 499–523. (doi:10.1163/15683908X399554)

221. Erhart EM, Overdorff DJ. 1998 Infanticide in *Propithecus diadema edwardsi*: An evaluation of the sexual selection hypothesis. *Int J Primatol* 19, 73–81. (doi:10.1023/A:1020306910493)

222. Wright PC. 1995 Demography and life history of free-ranging *Propithecus diadema edwardsi* in ranomafana national park, madagascar. *Int J Primatol* 16, 835–854. (doi:10.1007/BF02735722)

223. Ramsay MS, Morrison B, Stead SM. 2020 Infanticide and partial cannibalism in free-ranging Coquerel’s sifaka (*Propithecus coquereli*). *Primates* 61, 575–581. (doi:10.1007/s10329-020-00828-z)

224. Jolly A *et al.* 2000 Infant Killing, Wounding and Predation in Eulemur and Lemur. *Int J Primatol* 21, 239–248. (doi:https://doi.org/10.1023/A:1005467411880)

225. Andrews J. 1998 Infanticide by a female black lemur, *Eulemur macaco*, in disturbed habitat on Nosy Be, north-western Madagascar. *Folia Primatologica* 69, 14–17. (doi:10.1159/000052694)

226. Charpentier MJE, Drea CM. 2013 Victims of infanticide and conspecific bite wounding in a female-dominant primate: A long-term study. *PLoS One* 8. (doi:10.1371/journal.pone.0082830)

227. Kittler K, Dietzel S. 2016 Female infanticide and female-directed lethal targeted aggression in a group of ring-tailed lemurs (*Lemur catta*). *Primate Biol* 3, 41–46. (doi:10.5194/pb-3-41-2016)

228. Pereira MichaelE, Weiss MarkL. 1991 Female mate choice, male migration, and the threat of infanticide in ringtailed lemurs. *Behav Ecol Sociobiol* 28, 141–152. (doi:10.1007/BF00180991)

229. Vick LG, Pereira ME. 1989 Episodic targeting aggression and the histories of Lemur social groups. *Behav Ecol Sociobiol* 25, 3–12. (doi:10.1007/BF00299705)

230. Hood LC. 1994 Infanticide among ringtailed lemurs (*Lemur catta*) at Berenty Reserve, Madagascar. *Am J Primatol* 33, 65–69. (doi:10.1002/ajp.1350330107)

231. Ichino S. 2005 Attacks on a wild infant ring-tailed lemur (*Lemur catta*) by immigrant males at Berenty, Madagascar: Interpreting infanticide by males. *Am J Primatol* 67, 267–272. (doi:10.1002/ajp.20183)

232. Rasoloharijaona S, Rakotosamimanana B, Zimmermann E. 2000 Infanticide by a male Milne-Edwards’ sportive lemur (*Lepilemur edwardsi*) in Ampijoroa, NW-Madagascar. *Int J Primatol* 21, 41–45. (doi:10.1023/A:1005419528718)

233. Sushadi PS, WIRDATETI W, Phadmacanty NLPR, Wahyudin M. 2021 Short Communication: Infanticide of Javan slow loris (*Nycticebus javanicus*) in captivity. *Biodiversitas* 22, 1606–1611. (doi:10.13057/biodiv/d220403)

234. Grace F, Lukas KE, Kuhar C, Dennis PM. 2014 A retrospective review of mortality in lorises and pottos in North American zoos, 1980-2010. *Endanger Species Res* 23, 205–217. (doi:10.3354/esr00568)

235. Kenyon M, Phoung NT, Binh VT, Cronin A. 2023 The development of care protocols for pygmy lorises (*Xanthonycticebus pygmaeus*) from 2008 to 2023 at the Dao Tien Endangered Primate Species Centre, Cat Tien National Park, Vietnam. *Vietnamese Journal of Primatology*. 3.

236. Cäsar C, Franco ES, de Castro Nogueira Soares G, Young RJ. 2008 Observed case of maternal infanticide in a wild group of black-fronted titi monkeys (*Callicebus nigrifrons*). *Primates* 49, 143–145. (doi:10.1007/s10329-007-0067-9)

237. Roberts MILES. 1994 Growth, Development, and Parental Care in the Western Tarsier (*Tarsius bancanus*) in Captivity: Evidence for a ‘Slow’ Life-History. and Nonmonogamous Mating System. *Int J Primatol*. 15.

238. Gursky-Doyen S. 2011 Infanticide by a male spectral tarsier (*Tarsius spectrum*). *Primates* 52, 385–389. (doi:10.1007/s10329-011-0264-4)
